# Life Cycle Assessment-Based Comparative Study between High-Yield and “Standard” Bottom-Up Procedures for the Fabrication of Carbon Dots

**DOI:** 10.3390/ma15103446

**Published:** 2022-05-11

**Authors:** Sónia Fernandes, Joaquim C. G. Esteves da Silva, Luís Pinto da Silva

**Affiliations:** 1 Chemistry Research Unit (CIQUP), Institute of Molecular Sciences (IMS), Faculty of Sciences of University of Porto, R. Campo Alegre 697, 4169-007 Porto, Portugal; soniaf91991@gmail.com (S.F.); jcsilva@fc.up.pt (J.C.G.E.d.S.); 2 LACOMEPHI, GreenUPorto, Department of Geosciences, Environmental and Territorial Planning, Faculty of Sciences of University of Porto, R. Campo Alegre 697, 4169-007 Porto, Portugal

**Keywords:** life cycle assessment, carbon dots, bottom-up synthesis, high-yield synthesis, sustainability

## Abstract

Carbon dots (CDs) are carbon-based nanomaterials with remarkable properties that can be produced from a wide variety of synthesis routes. Given that “standard” bottom-up procedures are typically associated with low synthesis yields, different authors have been trying to devise alternative high-yield fabrication strategies. However, there is a doubt if sustainability-wise, the latter should be really preferred to the former. Herein, we employed a Life Cycle Assessment (LCA) approach to compare and understand the environmental impacts of high-yield and “standard” bottom-up strategies, by applying different life cycle impact assessment (LCIA) methods. These routes were: (1) production of hydrochar, via the hydrothermal treatment of carbon precursors, and its alkaline peroxide treatment into high-yield CDs; (2) microwave treatment of carbon precursors doped with ethylenediamine; (3) and (6) thermal treatment of carbon precursor and urea; (4) hydrothermal treatment of carbon precursor and urea; (5) microwave treatment of carbon precursor and urea. For this LCA, four LCIA methods were used: ReCiPe, Greenhouse Gas Protocol, AWARE, and USEtox. Results identified CD-5 as the most sustainable synthesis in ReCiPe, Greenhouse Gas Protocol, and USEtox. On the other hand, in AWARE, the most sustainable synthesis was CD-1. It was possible to conclude that, in general, high-yield synthesis (CD-1) was not more sustainable than “standard” bottom-up synthesis, such as CD-5 and CD-6 (also with relatively high-yield). More importantly, high-yield synthesis (CD-1) did not generate much lower environmental impacts than “standard” approaches with low yields, which indicates that higher yields come with relevant environmental costs.

## 1. Introduction

Carbon Dots (CDs) consist of fluorescent nanomaterials based on carbon. These possess a near-spherical shape and have either an amorphous or nanocrystalline core and a surface where different functional groups (alcohols, amines, and carboxylic acids, for example) can be found [1,2,3].

CDs possess various attractive features, such as strong luminescence [4,5], good physical–chemical and photochemical stability [6,7,8], water solubility [6], biocompatibility [9,10,11] and low toxicity [5]. These remarkable characteristics have attracted the research community, which is focused on developing several practical applications for CDs, such as in light-emitting devices [12,13,14], sensing [14,15,16,17,18], bioimaging [19,20,21], photocatalysis [22], drug delivery [23], solar cells [24] and in photodynamic therapy [25,26]. Beyond this, CDs can be synthesized using a wide variety of precursors without sophisticated equipment through different bottom-up strategies, such as solvothermal, hydrothermal, thermal, or microwave treatment of organic molecules [24,27,28,29,30,31]. While CDs can be generated from a wide variety of organic molecules as carbon sources, most of them can be expensive, as well as cause significant challenges to the environment and human health due to their use and/or synthesis [32].

One problem very present in CD development is related to their very low synthesis yields, which are generally equal to or below ~10% [33,34,35]. This can impair the production of CDs on a large-scale, as well as their future industrial applications. However, this problem can be solved if a way is found to convert the large amounts of solid carbon material, which are a major by-product of current synthesis strategies, into CDs.

As a matter of fact, some authors are already focusing on this topic [36] and they were able to produce CDs in high yield, achieving a synthesis yield of circa 20–40%. Despite these good results, the importance of these studies in the development of new CDs is still not validated in terms of efficiency, sustainability, and potential environmental impacts. To establish a suitable synthetic strategy for the sustainable fabrication of nanomaterials it is crucial to know this type of information, especially when the fabrication stage of nanomaterials was identified as an environmental concern [37]. In fact, some studies have defined that, during the life cycle of nanomaterials, the reagents and the energy required for the synthesis can contribute considerably to the environmental impacts [38,39]. In this sense, it is fundamental that before moving on to the industrial production of nanomaterials, the available synthesis routes are evaluated regarding their environmental impacts and sustainability to achieve the best alternatives for cleaner production.

Thus, our goal is to assess the environmental impacts associated with different synthesis route strategies of CDs, including high-yield and “standard” bottom-up synthesis. The “standard” bottom-up syntheses are related to either thermal or microwave-based heating of smaller organic molecules in powder form (calcination) or in solution (hydrothermal or solvothermal). These types of approaches can achieve a wide range of synthesis yields, albeit typically with lower yields. On the other hand, high-yield synthesis adds complexity to the synthesis procedures to ensure higher synthesis yields, by different approaches such as using a mixture of salts during thermal treatment, [40] or performing a subsequent alkaline peroxide treatment of the solid carbon by-product [36]. According to a previous study [41] that compared two high-yield syntheses, it was concluded that the procedure of an alkaline peroxide treatment of hydrochar [36] was more sustainable than a thermal treatment of carbon precursors mixed in a eutectic mixture of salts [40]. For this reason, the present work used the study [36] as the representative high-yield synthesis with the best environmental performance. In their turn, three studies [27,29,33] were considered where different “standard” bottom-up procedures (hydrothermal, microwave, and calcination) were performed. To achieve this aim, a life-cycle assessment (LCA) approach in order to quantify the environmental impacts of a given system during its life cycle [42,43,44] will be employed. Our LCA study will consider different parameters to evaluate the environmental impacts of the syntheses, for this, four distinct life cycle impact assessment (LCIA) methods will be used that specifically assess general parameters, CO_2_ emissions, water consumption, and toxicity. This tool (LCA) has already been used to evaluate the environmental impacts related to different types of engineered nanomaterials, such as carbon nanotubes [45,46,47,48], copper nanoparticles [49], graphene oxide [50], silver nanoparticles [39,51], nanocellulose [52], TiO_2_ nanoparticles [53], and even CDs [27,29,33,41,54]. Therefore, we expect to identify the synthesis route that is more sustainable and the most crucial parameters associated with the environmental impacts. More importantly, we want to evaluate if the higher synthesis yields of high-yield synthesis [36,40] can offset the expected higher environmental impacts associated with their complexity and added steps of high-yield synthesis, or if the simplicity of “standard” bottom-up procedures can lead to lower environmental impacts that can justify sustainability-wise lower synthesis yields.

## 2. Materials and Methods

The experimental section in this study is divided into four subsections: Scope and System Boundaries (2.1), Synthesis Routes (2.2), Life Cycle Inventory Data (2.3), and Environmental Impact Assessment (2.4).

### 2.1. Scope and System Boundaries

The present study consists of a cradle-to-grave LCA that aims for the evaluation of potential environmental impacts through their quantification and comparison, the assessment of the differences between these six types of CDs syntheses using different methods in these analyses, as well as the evaluation of two types of disposal scenarios. For this work, the laboratory-scale manufacturing stage of target nanoparticles was considered, as well as the direct emissions from CD production and indirect impacts related to the upstream resource extraction and energy generation.

**Scheme 1:**
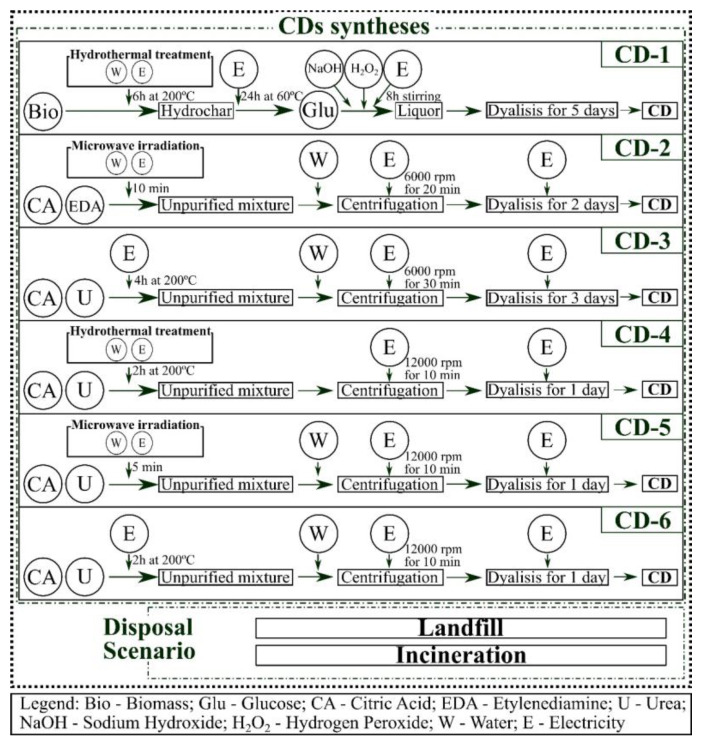
Diagram for the six routes of Carbon Dot (CD) production and two disposal scenarios.

This study uses six different synthesis routes for the fabrication of CDs [27,29,33,36] that englobe high-yield synthesis and different “standard” bottom-up synthesis strategies such as hydrothermal, microwave-assisted, and calcination, which are described in detail below (Scheme 1).

For this work, the environmental impacts were analyzed by using a weight-based functional unit of 1 kg, which means that 1 kg of produced CDs was considered. In this sense, it is possible to compare an equivalent amount of nanomaterials through the synthesis yield [55,56]. The synthesis yield of each CD under study can be viewed in Table 1.

### 2.2. Synthesis Routes

In this study, as mentioned above, six different syntheses of CDs [27,29,33,36] were compared, in which the used materials and electricity can be viewed in Table S1. CDs 1, 2, 3, 4, 5, and 6 produced by the synthesis are named CD-1, CD-2, CD-3, CD-4, CD-5, and CD-6, respectively. CD-1 [36] is used as a representative for high-yield synthesis, while other CDs are employed as representatives for different approaches of “standard” bottom-up methods.

The synthesis route for CD-1 begins with hydrothermal treatment of glucose for 6 h at 200 °C. Posteriorly, it follows the centrifugation to separate the suspension and the obtained hydrochar. This was then dried for 24 h at 60 °C in an oven. Finally, an alkaline peroxide treatment of the dried hydrochar was performed for 8 h, in which the hydrochar was dispersed into diluted NaOH solution as H_2_O_2_ was added. Then, this mixture was stirred for 8 h at room temperature and the pure CDs were obtained after 5-day dialysis to remove salts and other molecular impurities [36].

In CD-2 [33], the synthesis route begins with mixing 0.5 g of citric acid (CA) and 556 µL of Ethylenediamine (EDA) in 5 mL of deionized water. Then, a microwave treatment of this solution was performed for 10 min. Posteriorly, to remove unsuspended and insoluble aggregates, the synthesized CDs were suspended in water, and then the solution was purified by centrifugation for 20 min at 6000 rpm to eliminate impurities. Finally, the sample was redispersed in 10 mL of deionized water and then purified by dialysis for 48 h. The purified sample was dried at 80 °C to evaporate the water of the sample.

In the synthesis route for CD-3 [27], 2.5 g of citric acid and urea (U) was prepared with a proportion of 3:1 in a beaker. Then, this mixture was heated for 4 h at 200 °C in an oven. Later, the synthesized CDs were suspended in water and then the solution was purified by centrifugation for 30 min at 6000 rpm to eliminate impurities. Finally, the CDs were purified by dialysis for three days and then the sample was freeze-dried, in which the resulting powder was stored for further use.

For CD-4 [29], the synthesis route begins with mixing 0.75 g of CA and 0.25 g of U in 5 mL of deionized water. Then, this mixture was placed in a Teflon-lined reactor and was heated for 2 h at 200 °C in an oven. Posteriorly, the synthesized CDs were suspended in 5 mL of deionized water and purified by centrifugation for 10 min at 12,000 rpm to remove impurities. Finally, the sample was purified through a 24 h dialysis process.

In CD-5 [29], the synthesis begins by mixing 0.75 g of CA and 0.25 g of U in 5 mL of deionized water. Then, this mixture was placed in a glass beaker and subjected to a microwave treatment for 5 min. Later, the synthesized CDs were suspended in 5 mL of deionized water and purified by centrifugation for 10 min at 12,000 rpm to remove impurities. Lastly, the sample was purified through a dialysis process for 24 h.

In the synthesis route for CD-6 [29], 0.75 g of CA and 0.25 g of U were mixed in a glass Petri box and heated for 2 h at 200 °C in an oven. Then, the synthesized CDs were suspended in 5 mL of deionized water and purified by centrifugation for 10 min at 12,000 rpm to remove impurities. Finally, the sample was purified through a 24 h dialysis process.

### 2.3. Life Cycle Inventory Data

The evaluation of environmental impacts of CDs syntheses was based on inventory data from laboratory-scale synthesis procedures, the foreground data. This consists of raw materials (chemicals) and electricity (heating plate, oven, microwave, and centrifuge) used in the synthesis procedures (primary data). The foreground system included in this study was modeled from the data present in the Ecoinvent^®^ 3.5 database, where GLO stands for global, RER for regional market for Europe, and RoW for Rest-of-World. In this sense, the amount of chemicals and electricity used, as well as the data used from Ecoinvent^®^ 3.5 database are described in Table S1.

According to the Ecoinvent^®^ 3.5 database, for electricity, the dataset used is related to the available electricity data on the medium voltage level in Europe in 2014. The electricity considered, as referred above, consists of what is used in the heating plate, oven, microwave, and centrifuge. For CD-2, CD-3, CD-4, CD-5, and CD-6 the above were considered as the equipment identified in the studies [27,29,33]. On the other hand, in CD-1, since the authors of this study did not identify the used equipment, we decided to apply standard plates and ovens. In this sense, the Normax Nx1200 Analogical magnetic stirrer with heating was considered, which has a power consumption of 500 W. The oven considered was the DRY-Line^®^ Prime from VWR, with 12 A and a power supply in AC (single phase) of 230 V, which can achieve with a maximum power factor (1) a power consumption maximum of 2760 W. In CD-3, for dialysis, the heating plate referred to above was considered, and was also considered as the equipment used for the centrifugation in CD-2, CD-4, CD-5, and CD-6.

Regarding the disposal scenarios and the Ecoinvent^®^ 3.5 database, two possible scenarios were considered, incineration and landfill.

### 2.4. Environmental Impact Assessment

This LCA study used a cradle-to-grave approach, from the production of precursor materials to the disposal scenario, also including the fabrication of CDs. The environmental impacts were modeled by four different LCIA methods, ReCiPe 2016 V1.03 endpoint method, Greenhouse Gas Protocol V1.02 method, AWARE (Available Water Remaining) V1.02 method, and USEtox 2 V1.00 method. This LCA study was performed with SimaPro 9.0.0.48 software.

ReCipe 2016 method, Hierarchist version [56] evaluates the environmental impacts in three categories of potential impacts (Human Health, Ecosystems, and Resources), which posteriorly are subdivided into subcategories. In the Human Health (HH) category, Global Warming–Human Health (GW–HH), Stratospheric ozone depletion (SO), Ionization Radiation (IR), Ozone formation–Human Health (OF), Fine Particulate Matter formation (FPM), Human Carcinogenic toxicity (HC), Human Non-Carcinogenic toxicity (HNC) and Water Consumption–Human Health (WC–HH) were assessed. On the other hand, in the Ecosystems ® category, the potential environmental impacts evaluated were Global Warming–Terrestrial Ecosystems (GW–TE), Global Warming–Freshwater Ecosystems (GW–FE), Ozone Formation–Terrestrial Ecosystems (OF–TE), Terrestrial acidification (TA), Freshwater Eutrophication (FE), Marine Eutrophication (ME), Terrestrial EcoToxicity (TET), Freshwater EcoToxicity (FET), Marine EcoToxicity (MET), Land Use (LU), Water Consumption–Terrestrial Ecosystem (WC–TE) and Water Consumption–Aquatic Ecosystems (WC–AE). Lastly, in the Resourc®(R) category, Mineral Resource scarcity (MR) and Fossil Resource scarcity (FR) were assessed.

The Greenhouse Gas Protocol method evaluates the CO_2_ emissions through four categories, Fossil CO_2_, Biogenic CO_2_, CO_2_ from land transformation, and CO_2_ uptake. In Fossil CO_2_, the fossil-based carbon, more specifically the carbon originating from fossil fuels is assessed. On the other hand, in the Biogenic CO_2_ category, the biogenic carbon is considered, which is the carbon originating from biogenic sources. In the CO_2_ from land transformation category, only the direct impacts are assessed and, in turn, in the carbon uptake category, the carbon dioxide stored in plants and trees as they grow is considered.

The AWARE method assesses the water consumption impact in an LCA, more specifically it considers the potential of water deprivation, either to humans or ecosystems.

Finally, the USEtox 2 method evaluates the human and ecotoxicological impacts of chemicals in two categories, Human Health and Ecosystems. In the Human Health category, Human toxicity, cancer and Human toxicity, non-cancer are assessed. On the other hand, in the Ecosystems category, freshwater ecotoxicity is considered.

## 3. Results and Discussion

This section is divided into four subsections related to the methods used in the present LCA: ReCiPe method (3.1), Greenhouse Gas Protocol method (3.2), AWARE method (3.3), and USEtox method (3.4).

In this LCA, a comparison of potential environmental impacts, considering a weight-based functional unit of 1 kg of CDs was performed. However, our intention was to compare, in each method, the contributions of the different impact categories of the input involved in each synthesis and with each other. In this sense, it is not intended to make quantitative appreciations of the environmental impacts of each material input. As indicated before, CD-1 is used as a representative for high-yield synthesis, while CD-2 to CD-6 are representatives of different alternatives for “standard” bottom-up methods. More importantly, these latter CDs represent a wide range of synthesis yields (Table 1).

### 3.1. Impact Assessment by ReCiPe Method

The individual results regarding the associated environmental impacts obtained for CD-1, CD-2, CD-3, CD-4, CD-5, and CD-6 can be viewed in Figure S1. According to the input of each synthesis under the study, it was possible to verify that the major contributor in all subcategories was CA for CD-4 (Figure S1D) with environmental impacts varying between 67 and 99%. However, for the remaining syntheses in almost all the subcategories, electricity was the higher contributor in CD-1 (with contributions between 56 and 96%), CA was responsible for most of the impacts in CD-2 (between 41 and 94%), CD-3 (between 53 and 95%), CD-5 (between 48 and 95%), and CD-6 (between 54 and 95%). In this sense, for CD-1 (Figure S1A), the carbon precursor (glucose) constitutes the highest contribution to environmental impacts (circa 61%) in Marine Eutrophication (ME) and Land Use (LU). In CD-2 (Figure S1B), electricity was the major contributor (circa 49%) in Ionizing Radiation (IR) and EDA was responsible for most of the impacts in ME and Fossil Resource scarcity (FR), contributing by approximately 63% and 67%, respectively. For CD-3 (Figure S1C) and CD-6 (Figure S1F), electricity was the highest contributor to IR and Freshwater Eutrophication (FE), being responsible for circa 80% (CD-3: 84% and CD-6: 83%) and 50% (CD-3: 52% and CD-6: 51%) of impacts. In CD-5 (Figure S1E), electricity was responsible for most of the impacts in the IR subcategory, contributing to 82% of the environmental impacts. It is worth mentioning that for this CD, in the FE subcategory, CA and electricity have similar contributions to the environmental impacts, with 48% and 47%, respectively. On the other hand, for all the syntheses in the study, water appears to have quite negligible impacts in almost all the subcategories. In CD-1, hydrogen peroxide also seems to have negligible environmental impacts.

**Figure 1:**
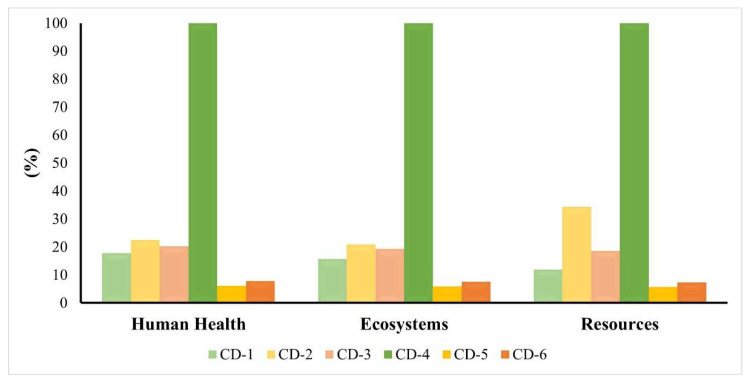
Relative environmental impacts of syntheses under study, applying ReCiPe method. Green scale refers to hydrothermal treatments; Yellow scale refers to microwave-assisted treatments; Orange scale refers to calcination treatments.

The comparison of associated environmental impacts for the six syntheses can be observed in Figure 1, in which all environmental impact subcategories are included in only three main categories (Human Health, Ecosystems, and Resources). The first conclusion was that synthesis 5 (CD-5) is associated with lower environmental impacts and synthesis 4 (CD-4) with the highest impacts. In this sense, it is possible to understand that hydrothermal treatment has more impacts than microwave treatment. However, in CD-1 (in which hydrothermal treatment was also performed), the environmental impacts are expressively lower than in CD-4. This can be attributed to the distinct synthesis yields, which are 40.1% for CD-1 and 1.8% for CD-4. It is worth mentioning that, only in CD-2 is it possible to observe expressively higher impacts in the Resources category than in the other categories (difference of 12% and 14% of impacts, respectively, with Human Health and Ecosystems). One possible explanation for this can be attributed to EDA, which as we mentioned above, is the highest contributor in the FR subcategory, one of the two subcategories that are included in the Resources category.

It was also possible to observe from the previous figure that CD-1 does not have a lower environmental impact when compared to CD-2 and CD-3. However, there is a significant difference between the synthesis yield of these syntheses (Table 1). In this sense, it was verified that, between CD-1 and CD-2, the differences in potential impacts were 5% in Human Health and Ecosystems, and 22% in Resources. On the other hand, the variations in the contributions were 2% in Human Health, 4% in Ecosystems, and 7% in Resources, between CD-1 and CD-3. The major differences in amounts of inputs of these syntheses were in water and energy, in which CD-1 used less water than CD-2 and CD-3 (CD-1: 24.9 kg; CD-2: 77.9 kg; CD-3: 101.5 kg), and higher electricity in CD-1 when compared to the other syntheses (CD-1: 146.8 kWh; CD-2: 24.2 kWh; CD-3: 46.09 kWh). Considering the energy inputs to perform CD-1 it was verified that the higher input is related to the hydrothermal treatment and the drying process (82.8 kWh) by the oven. For the stirring step and dialysis, only 4 kWh and 60 kWh were needed. On the other hand, in CD-2 and CD-3, the higher energy input is related to dialysis (CD-2: 24 kWh; CD-3: 36 kWh). The remaining inputs are related to microwave (0.12 kWh) and centrifuge (0.06 kWh) in CD-2, and to the oven (10 kWh) and centrifuge (0.09 kWh) in CD-3. Thus, these smaller differences in potential environmental impacts between high-yield synthesis and “standard” bottom-up syntheses with low yield (less than 10%) suggest that the first may not offset the higher yield. Nevertheless, given that CD-1 spends a significant amount of energy when drying with an oven, employing a less energy-consuming strategy for this step might reduce the associated environmental costs in a relevant manner.

Once our LCA considers a cradle-to-grave approach, it is important to evaluate disposal scenarios of the synthesized CDs. These scenarios intend to assess a more advanced stage of the life cycle of these CDs, considering either incineration or landfill disposal.

In incineration, for each synthesis, it was possible to understand that the synthesis has more environmental impacts than the disposal scenario in all the subcategories (Figure S2). Thus, the potential impacts of synthesis vary between 91–100% for CD-1, 90–100% for CD-2, 89–100% for CD-3, 97–100% for CD-4, 71–100% for CD-5, and 76–100% for CD-6. It is worth mentioning that the incineration scenario has quite negligible impacts with the exception of the FET, MET, and HNC subcategories in CD-1 (respectively, 9%, 9%, and 7%), CD-2 (respectively, 10%, 9%, and 6%), CD-3 (respectively, 11%, 10%, and 7%), and CD-4 (respectively, 3%, 3%, and 2%). On the other hand, the exceptions for CD-5 and CD-6 were the GW–HH (2% for both CDs), GW–TE (2% for both CDs), GW–FE (2% for both CDs), SO (CD-5: 2%), FET (CD-5: 29% and CD-6: 24%), MET (CD-5: 27% and CD-6: 22%), HC (CD-5: 4% and CD-6: 3%), and HNC (CD-5: 20% and CD-6: 16%) subcategories.

**Figure 2:**
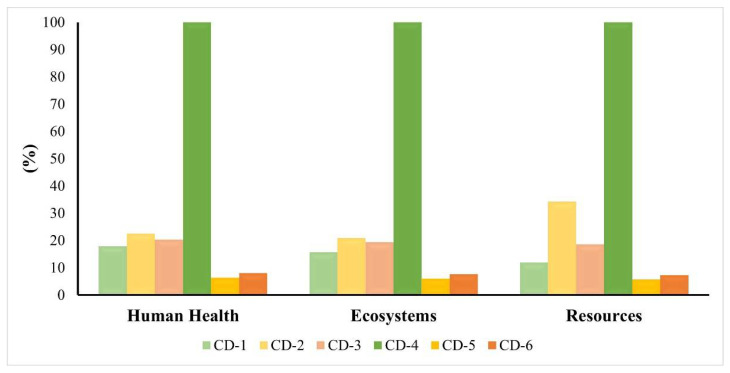
Relative environmental impacts of syntheses under study for incineration disposal scenario, applying ReCiPe method. Green scale refers to hydrothermal treatments; Yellow scale refers to microwave-assisted treatments; Orange scale refers to calcination treatments.

Comparing all the syntheses (Figure 2) for the incineration scenario it was possible to understand that CD-5 is associated with lower environmental impacts and CD-4 with the highest impacts. Once again, it is possible to verify that CD-2 has higher impacts in the Resources category than the other categories (differences of 12% with HH and 14% with E).

For the landfill scenario, it was also verified that the synthesis is responsible for more impacts than the disposal scenario in all the syntheses under study (Figure S3). In this sense, the synthesis potential environmental impacts vary between 87–100% for CD-1, 85–100% for CD-2, 84–100% for CD-3, 96–100% for CD-4, 61–100% for CD-5, and 67–100% for CD-6. For all the syntheses it was possible to understand that the disposal scenario has quite negligible impacts with some exceptions. In CD-1 and CD-3, these were in ME (CD-1: 4% and CD-3: 3%), FET (CD-1: 14% and CD-3: 16%), MET (CD-1: 13% and CD-3: 15%), and HNC (CD-1: 12% and CD-3: 13%). On the other hand, for CD-2 and CD-4, the exceptions were in FET (CD-2: 15% and CD-4: 4%), MET (CD-2: 14% and CD-4: 4%), and HNC (CD-2: 11% and CD-4: 3%). Finally, GW–HH (3% for both CDs), GW–TE (3% for both CDs), GW–FE (3% for both CDs), ME (CD-5: 9% and CD-6: 7%), FET (CD-5: 39% and CD-6: 33%), MET (CD-5: 38% and CD-6: 32%), HC (CD-5: 2%), and HNC (CD-5: 33% and CD-6: 28%) were the exceptions in CD-5 and CD-6.

**Figure 3:**
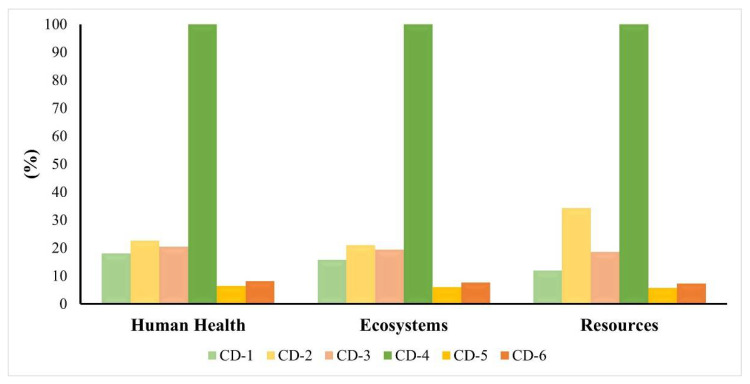
Relative environmental impacts of syntheses under study for landfill disposal scenario, applying ReCiPe method. Green scale refers to hydrothermal treatments; Yellow scale refers to microwave-assisted treatments; Orange scale refers to calcination treatments.

For the landfill scenario, considering all the syntheses (Figure 3), it was possible to corroborate once again that CD-5 has the lowest environmental impacts and CD-4 is related to the highest impacts. In this disposal scenario, it also verifies that CD-2 has higher impacts in the Resources category than the others.

**Figure 4:**
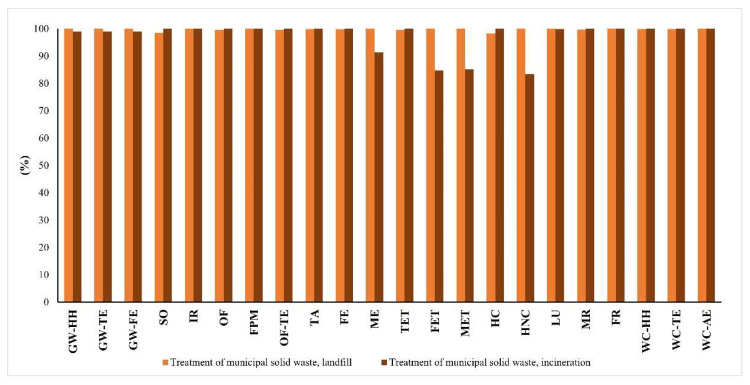
Comparative relative environmental impacts of CD-5 for incineration and landfill disposal scenario, applying ReCiPe method.

For a better understanding of the relative environmental impacts of different disposal scenarios, we compared the environmental impacts for CD-5 (the route with lower impacts) when considering both incineration and landfill. It was observed that incineration has slightly lower impacts than landfill in the three main categories (Figure S4). However, the evaluation of the subcategories allows understanding that incineration has significantly lower impacts in ME, FET, MET, and HNC with differences of 9%, 15%, 15%, and 17%, respectively (Figure 4). It is worth mentioning that, in the Resources category (MR and FR subcategories), landfill and incineration have almost the same environmental impacts.

### 3.2. Impact Assessment by Greenhouse Gas Protocol Method

The individual results regarding the associated environmental impacts obtained for all the syntheses with the Greenhouse Gas Protocol method can be observed in Figure S5. It can be seen that, when considering each input of the syntheses under study, the major contributor to the environmental impacts was electricity in CD-1 (with contributions of between 55 and 97%) and CA in CD-2 (contributions varying from 50–92%), CD-3 (contributions varying from 51–93%), CD-4 (contributions varying from 83–99%), CD-5 (contributions varying from 56–94%), and CD-6 (contributions varying from 53–93%). It is worth mentioning that in CD-1 (Figure S5A), the contributions of electricity and glucose are slightly similar (differences of 10%) in the CO_2_ uptake category. In CD-2 (Figure S5B), electricity and EDA have a slightly higher contribution to the impacts in the Fossil and Biogenic CO_2_ categories, in which EDA is higher in the first and electricity in the second category. On the other hand, in CD-3 (Figure S5C), electricity has a greater impact on Biogenic CO_2_ than in the other three categories but is still smaller than CA (a difference of 4%). Finally, in CD-4, CD-5, and CD-6, electricity has a higher impact on Biogenic CO_2_ (CD-4: 5%, CD-5: 42% and CD-6: 45%) and urea in Fossil CO_2_ (CD-4: 15%, CD-5 and CD-6: 12%), with CA still being responsible for most impacts in both categories. It is important to note that, the relative contributions of water in all the syntheses and hydrogen peroxide in CD-1 appear to be quite negligible.

**Figure 5:**
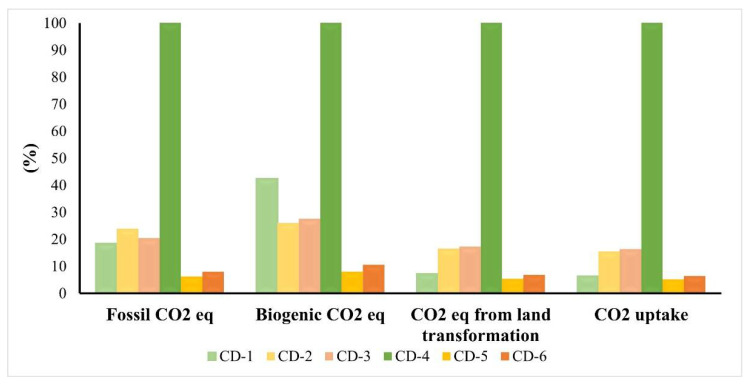
Relative environmental impacts of syntheses under study, applying Greenhouse Gas Protocol method. Green scale refers to hydrothermal treatments; Yellow scale refers to microwave-assisted treatments; Orange scale refers to calcination treatments.

The comparison of all the syntheses (Figure 5) allowed us to understand that CD-5 is associated with lower environmental impacts and CD-4 has the highest impacts, which also permitted us to verify that the hydrothermal treatment has more impacts than the microwave treatment. Despite a hydrothermal treatment being performed in CD-1, the potential impacts were significantly lower than in CD-4 due to the differences in the synthesis yield. It is noteworthy that the environmental impacts for CD-1 were considerably higher in the Biogenic CO_2_ category and drastically lower in CO_2_ uptake. This analysis also allowed us to understand that the Biogenic CO_2_ category in this method is a key category in CD syntheses since the relative impacts were higher when compared with the other categories, mainly in CD-1 (differences between 24% and 36%).

To evaluate the entire life cycle of the synthesized CDs an assessment of incineration and landfill disposal scenarios was carried out.

For the incineration scenario, the results obtained for each synthesis can be observed in Figure S6. This analysis also allowed us to understand that the synthesis has more environmental impacts than the disposal scenario. The contributions of the synthesis vary between 91–100% in CD-1 (Figure S6A), 85–100% in CD-2 (Figure S6B), 86–100% in CD-3 (Figure S6C), 96–100% in CD-4 (Figure S6D), 64–100% in CD-5 (Figure S6E), and 70–100% in CD-6 (Figure S6F). In this sense, the impacts of incineration are quite negligible with the exception of Biogenic CO_2_ in CD-1 (9%), CD-2 (15%), CD-3 (14%), CD-4 (4%), CD-5 (2%), and CD-6 (2%). Fossil CO_2_ is also an exception in CD-5, with contributions of 36%, and in CD-6 with 30%.

**Figure 6:**
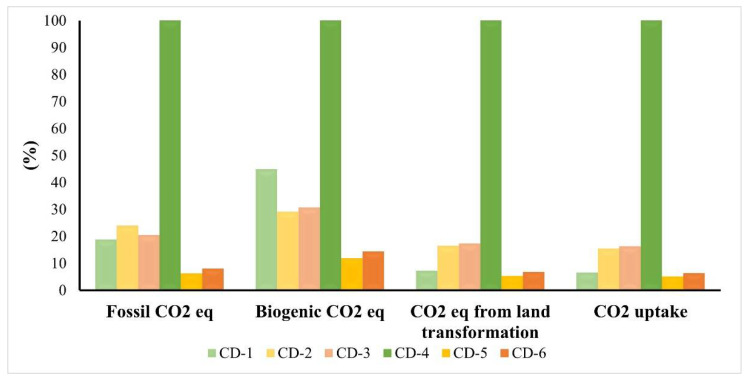
Relative environmental impacts of syntheses under study for incineration disposal scenario, applying Greenhouse Gas Protocol method. Green scale refers to hydrothermal treatments; Yellow scale refers to microwave-assisted treatments; Orange scale refers to calcination treatments.

Comparing all the syntheses for the incineration scenario (Figure 6) it was possible to verify that CD-5 has lower environmental impacts and CD-4 is associated with the highest impacts, as we observed in the ReCiPe method. It was possible to observe that, for all the CDs, in the Biogenic CO_2_ category the impacts are higher when compared with the other categories. These differences are expressively higher in CD-1, in which the contributions rise between 26% and 38%. That allowed us to understand that in this method the Biogenic CO_2_ was a key category in CD syntheses, since the impacts of incineration have greater contributions, mainly in CD-1.

On the other hand, for the landfill disposal scenario, the results obtained can be observed in Figure S7. It was possible to observe that, once again, the synthesis has higher impacts than the disposal scenario. Thus, the environmental impacts of synthesis range between 91–100% in CD-1 (Figure S7A), 86–100% in CD-2 (Figure S7B), 87–100% in CD-3 (Figure S7C), 96–100% in CD-4 (Figure S7D), 66–100% in CD-5 (Figure S7E), and 72–100% in CD-6 (Figure S7F). As a result, the contributions of the landfill scenario are quite negligible with the exception of Biogenic CO_2_ in all the syntheses (CD-1: 9%, in CD-2: 14%, in CD-3: 13%, in CD-4: 4%, in CD-5: 35%, and in CD-6: 29%).

**Figure 7:**
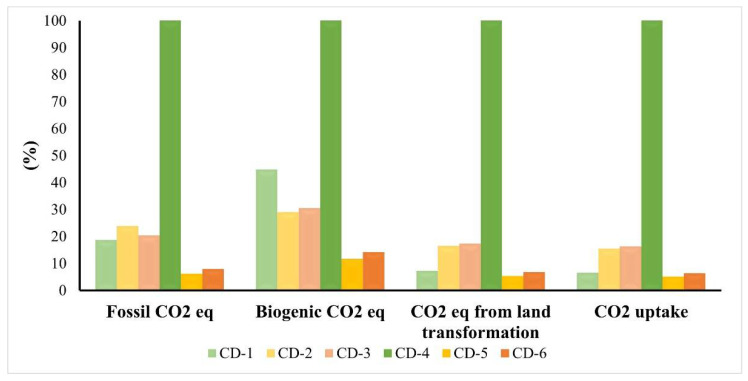
Relative environmental impacts of syntheses under study for landfill disposal scenario, applying Greenhouse Gas Protocol method. Green scale refers to hydrothermal treatments; Yellow scale refers to microwave-assisted treatments; Orange scale refers to calcination treatments.

The comparison of all the syntheses for the landfill disposal scenario (Figure 7) allowed us to understand that CD-5 has minor impacts and CD-4 has the highest environmental impacts. It is worth mentioning that, once again, in all the syntheses of CDs, the impacts were higher in the Biogenic CO_2_ category, especially in CD-1.

**Figure 8:**
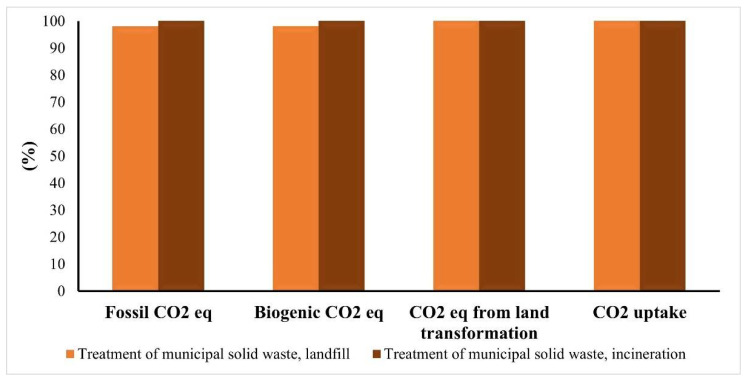
Relative environmental impacts of CD-5 for incineration and landfill disposal scenario, applying Greenhouse Gas Protocol method.

Considering the synthesis with lower impacts (CD-5), the disposal scenarios that were more sustainable were evaluated (Figure 8). In this sense, we verify that for CO_2_ from land transformation and CO_2_ uptake categories, the contributions of landfill and incineration have the same environmental impacts. However, in Fossil and Biogenic CO_2_, the landfill has slightly lower impacts than incineration (a difference of 2%). According to the previous method (ReCiPe), it was verified that for the Human Health and Ecosystem categories, incineration has slightly lower impacts. This allowed us to conclude that, for CD-5, the landfill has minor environmental impacts on CO_2_ emissions (Fossil and Biogenic) and incineration has lower impacts on Human Health and Ecosystems.

### 3.3. Impact Assessment by AWARE Method

The results obtained by this method for each synthesis under study can be observed in Figure S8. It was possible to observe that for water use, the major contributor was electricity in CD-1 (with contributions of 76%) and CA in the remaining syntheses (CD-2: 78%, CD-3: 80%, CD-4: 84%, CD-5: 81%, and CD-6: 80%). In relation to the other synthesis inputs, it was possible to verify a trend (from the highest to the lowest contributor) of electricity > glucose > NaOH > H_2_O_2_ > H_2_O in CD-1 (Figure S8A), CA > EDA > electricity > H_2_O in CD-2 (Figure S8B), CA > urea > electricity > H_2_O in CD-3 (Figure S8C), CA > urea > H_2_O > electricity in CD-4 (Figure S8D), and CA > urea > electricity > H_2_O in CD-5 (Figure S8E) and CD-6 (Figure S8F). In general, it was possible to understand that CD-2, CD-3, CD-5, and CD-6 appear to have a similar trend, in which we have a carbon precursor as the higher contributor, followed by the N-containing small organic molecules (EDA or urea) and then, electricity and water. It is worth mentioning that in CD-4 and CD-6, water appears to have negligible impacts (less than 1%).

**Figure 9:**
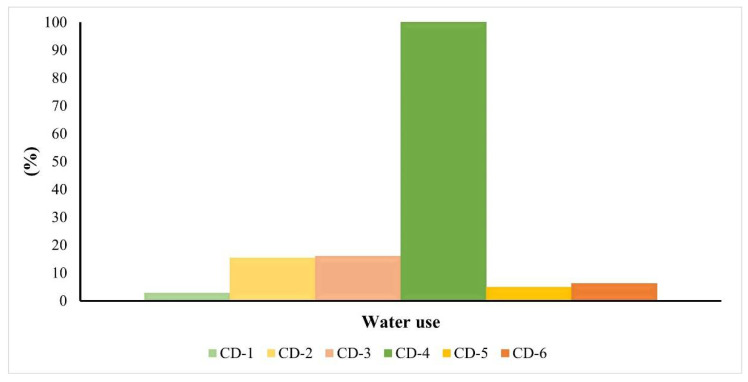
Relative environmental impacts of all syntheses under study, applying AWARE method. Green scale refers to hydrothermal treatments; Yellow scale refers to microwave-assisted treatments; Orange scale refers to calcination treatments.

Comparing all the syntheses under study (Figure 9), the first conclusion was that CD-1 is associated with lower environmental impacts and CD-4 with the highest impacts. These synthesis routes performed a hydrothermal treatment of the precursors, and the major difference is that CD-1 consists of a high-yield synthesis. Furthermore, the carbon precursor in these syntheses is different (CD-1: glucose and CD-4: citric acid), which can also explain the difference (97%) in the potential environmental impacts. It is worth mentioning that this method allowed us to understand that, when we focus on the potential of water deprivation, the high-yield synthesis (CD-1) is more sustainable.

The contribution of incineration and landfill disposal scenarios to the water consumption of the synthesized CDs was also evaluated.

For each synthesis under study, the analysis of the incineration scenario can be observed in Figure S9. In all the synthesized CDs, the synthesis was responsible for most of the environmental impacts, meaning the disposal scenario contributions were negligible. This method allowed us to understand that, for water use, the synthesis has an expressively higher contribution than incineration (CD-1: 99.81%, CD-2: 99.96%, CD-3: 99.97%, CD-4: 99.99%, CD-5: 99.89%, and CD-6: 99.91%).

**Figure 10:**
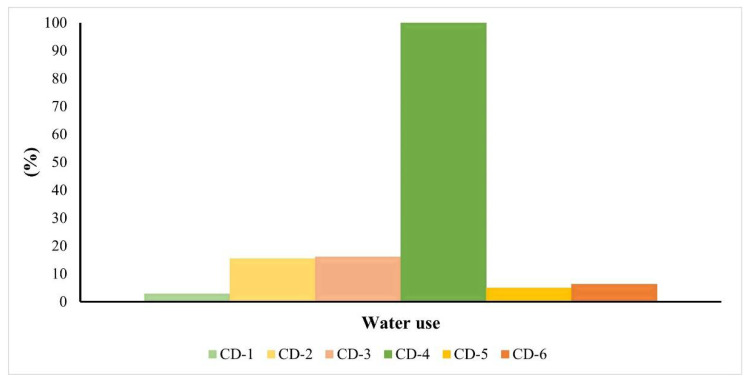
Relative environmental impacts of all the syntheses under study for incineration disposal scenario, applying AWARE method. Green scale refers to hydrothermal treatments; Yellow scale refers to microwave-assisted treatments; Orange scale refers to calcination treatments.

Comparing all the syntheses for the incineration scenario (Figure 10), we concluded that CD-1 has lower environmental impacts and CD-4 is associated with higher impacts. These syntheses have a difference of 97% in the potential impacts, despite both having a hydrothermal approach.

The evaluation of the landfill scenario of each synthesis under study can be observed in Figure S10. It was possible to conclude that, in all synthesized CDs, the contributions of the disposal scenario were quite negligible, meaning the synthesis step had more environmental impacts. In this sense, the synthesis was responsible for 99.95% in CD-1 (Figure S10A), 99.99% in CD-2 (Figure S10B), CD-3 (Figure S10C) and CD-4 (Figure S10D), 99.97% in CD-5 (Figure S10E), and 99.98% in CD-6 (Figure S10F).

**Figure 11:**
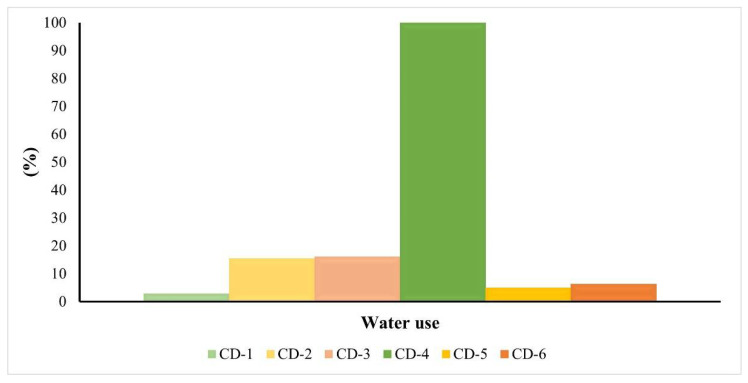
Relative environmental impacts of all syntheses for landfill disposal scenario, applying AWARE method. Green scale refers to hydrothermal treatments; Yellow scale refers to microwave-assisted treatments; Orange scale refers to calcination treatments.

In the evaluation of the landfill scenario, a comparison of all the syntheses under study was also performed (Figure 11). That allowed us to conclude that in this scenario CD-1 is also related to lower impacts and CD-4 with higher environmental impacts. Thus, this shows once again the sustainability of CD-1 regarding water deprivation.

**Figure 12:**
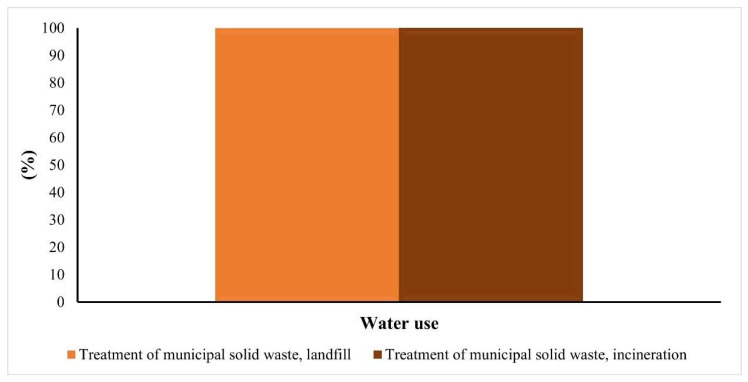
Relative environmental impacts of CD-1 for incineration and landfill disposal scenario, applying AWARE method.

According to the synthesis with lower impacts in this method, an evaluation of the disposal scenario (Figure 12) was performed. This analysis permitted us to conclude that no significant differences exist between the disposal scenarios, despite landfill having slightly lower environmental impacts than incineration (a difference of 0.14%). Therefore, as verified in the previous method (Greenhouse Gas Protocol method), the landfill scenario appears to be more sustainable regarding CO_2_ emissions and water consumption.

### 3.4. Impact Assessment by USEtox Method

The assessment of human and ecotoxicological impacts for each synthesis under study can be observed in Figure S11. This allowed us to conclude that the major contributor was electricity in CD-1 (with contributions between 80 and 93%) and CA in the remaining syntheses (CD-2: 44–61%, CD-3: 52–67%, CD-4: 87–93%, CD-5: 56–71%, and CD-6: 54–69%). In CD-1 (Figure S11A), it was possible to observe that glucose has the highest contribution to the Human Toxicity non-cancer category, being responsible for 16% of the impacts (a difference of 13% when compared to the other subcategories). For CD-2 it was verified that the highest contributor is not completely isolated from the other inputs, especially from EDA in Human Health (differences of 12%). It is still worth mentioning that, in this synthesis, EDA has a higher impact than electricity. In CD-3, a similitude of potential environmental impacts between CA and electricity exists in the Human Toxicity cancer subcategory (difference of 10%). For CD-4 (Figure S11D), in all subcategories, urea has more environmental impacts than electricity. On the contrary, in CD-3 (Figure S11C), CD-5 (Figure S11E) and CD-6 (Figure S11F), electricity was responsible for more environmental impacts than urea in all the subcategories. It is noteworthy that water appears to have quite negligible environmental impacts in all the syntheses, as compared with hydrogen peroxide in CD-1.

**Figure 13:**
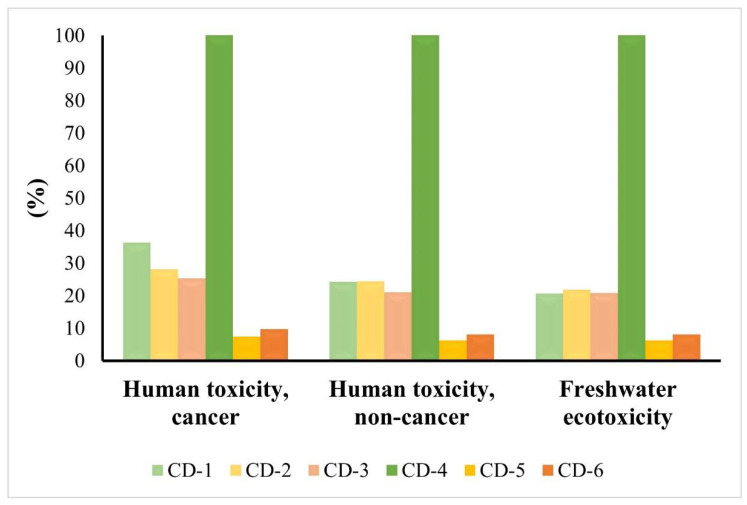
Relative environmental impacts of all syntheses under study, applying USEtox method. Green scale refers to hydrothermal treatments; Yellow scale refers to microwave-assisted treatments; Orange scale refers to calcination treatments.

The comparison of all syntheses under study (Figure 13) allowed us to conclude that CD-5 is associated with lower impacts and CD-4 with the highest environmental impacts. Thus, this method observed that, for toxicological impacts of chemicals, CD-5 appears to be more sustainable than the other syntheses, as we observed in the ReCiPE and Greenhouse Gas Protocol method.

For this method, the incineration and landfill disposal scenarios of the synthesized CDs were evaluated.

The results obtained for incineration in each synthesis under study can be observed in Figure S12. It was possible to observe that the synthesis was responsible for more environmental impacts than the disposal scenario. In this sense, the contributions of the synthesis varied between 94–99% in CD-1 (Figure S12A) and CD-3 (Figure S12C), 95–99% in CD-2 (Figure S12B), 99–100% in CD-4 (Figure S12D), 83–96% in CD-5 (Figure S12E), and 86–97% in CD-6 (Figure S12F). Thus, the potential impact of incineration appears to be quite negligible, especially in the Human Toxicity cancer subcategory for CD-1, CD-2, and CD-4 (contributions less than 1%). However, for CD-4, in the Human Toxicity non-cancer subcategory the impacts were also less than 1%. For CD-3, it was observed that the impacts were lower in the Human Health category (i.e., human toxicity with cancer and human toxicity without cancer subcategories) than in Freshwater ecotoxicity. It is worth mentioning that for CD-5 and CD-6, in all subcategories, the environmental impacts of incineration were lower, when compared with the previous syntheses. On the other hand, for CD-5 and CD-6, the difference in potential impacts between Human Health and Ecosystems was higher. That is expressively evident in the Freshwater ecotoxicity subcategory, in which incineration was responsible for 17% and 14% of environmental impacts, respectively, in CD-5 and in CD-6.

**Figure 14:**
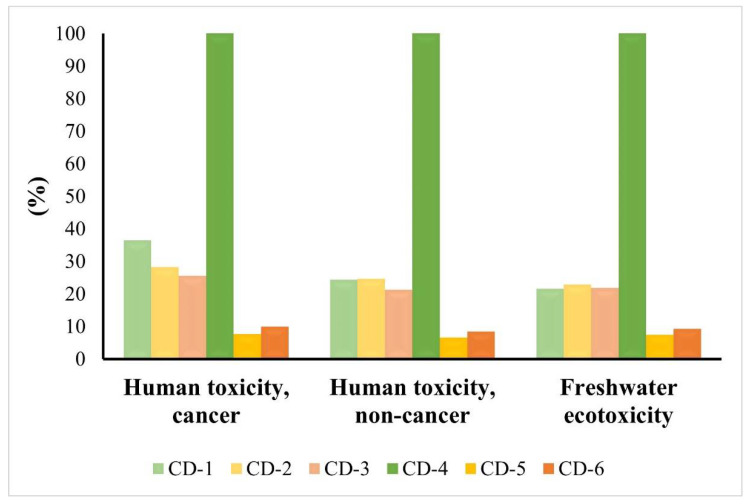
Relative environmental impacts of all the syntheses under study for incineration disposal scenario, applying USEtox method. Green scale refers to hydrothermal treatments; Yellow scale refers to microwave-assisted treatments; Orange scale refers to calcination treatments.

Comparing all the syntheses for the incineration scenario (Figure 14), it was possible to understand that CD-5 has lower environmental impacts and CD-4 has higher impacts. Furthermore, CD-1, CD-2, and CD-3 have higher impacts in the Human Health category than in the Ecosystem category. It is worth mentioning that CD-1 has expressively higher impacts on Human toxicity with cancer, with differences of 12% and 15% when compared to Human toxicity without cancer and Freshwater ecotoxicity, respectively. This allowed us to conclude that CD-1 presents less sustainability when we assessed the ecotoxicity of chemicals. That can be explained due to the alkaline peroxide treatment performed in this synthesis route.

On the other hand, the analysis of the landfill disposal scenario of each synthesized CD can be observed in Figure S13. It was possible to understand that, once again, the synthesis scenario is associated with higher environmental impacts than the disposal scenario. In this sense, the contributions of synthesis vary between 91–100% in CD-1 (Figure S13A), 92–99% in CD-2 (Figure S13B) and in CD-3 (Figure S13C), 98–100% in CD-4 (Figure S13D), 76–98% in CD-5 (Figure S13E), and 81–98% in CD-5 (Figure S13F). It is noteworthy that, in CD-1, CD-2, and CD-3, the impacts of the landfill scenario were quite negligible in the Human Toxicity with cancer subcategory. Whereas in the remaining subcategories the contributions were responsible for more than 2% of impacts. In CD-4, the contributions of the landfill category appear to be very insignificant in both subcategories of the Human Health category (less than 0.5% of impacts). Finally, in CD-5 and CD-6, the environmental impacts of the disposal scenario were lower in all subcategories when compared with the other synthesis. However, the difference in impacts between the Human Health subcategories and Ecosystems was higher than in the other syntheses. This is expressively evident in freshwater, where the contributions were 24% and 19% of impacts in CD-5 and CD-6, respectively.

**Figure 15:**
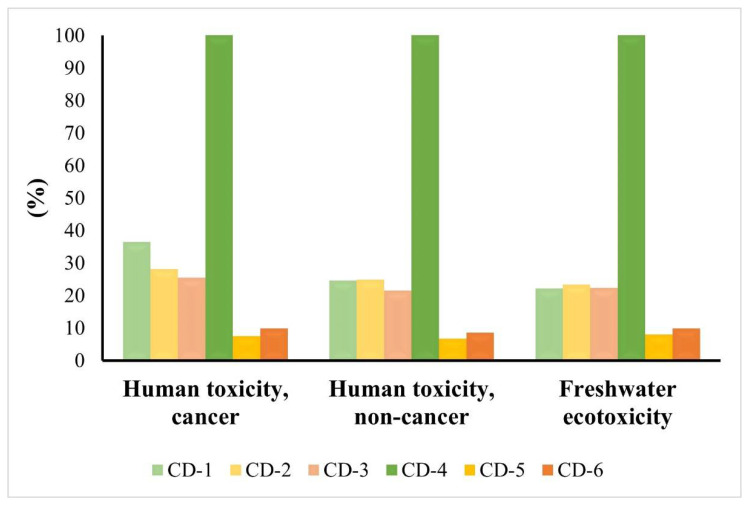
Relative environmental impacts of all syntheses for landfill disposal scenario, applying USEtox method. Green scale refers to hydrothermal treatments; Yellow scale refers to microwave-assisted treatments; Orange scale refers to calcination treatments.

The evaluation of all the syntheses in respect of the landfill scenario (Figure 15) allowed us to understand that CD-5 has lower impacts and CD-4 was associated with higher environmental impacts. Once again, it was verified that CD-1, CD-2, and CD-3 have higher impacts on Human Health than in the Ecosystem category. This is expressively evident in CD-1, where differences between both subcategories of Human Health and the Human toxicity, cancer with Freshwater ecotoxicity were 12% and 14%, respectively. Thus, once again, when we compared the impacts of CD-1 considering the other methods, it was concluded that CD-1 presents less sustainability related to the ecotoxicity of chemicals.

**Figure 16:**
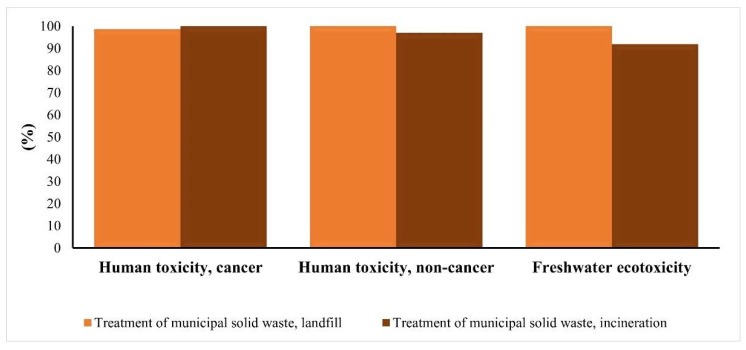
Relative environmental impacts of CD-5 for incineration and landfill disposal scenario, applying USEtox method.

Considering the synthesis with lower impacts in this method (CD-5), we verified that the incineration scenario has slightly lower environmental impacts than the landfill scenario (a difference of 0.9% for Human Health and 8.1% for Ecosystems), as is noted in Figure 16. This was also seen for the ReCiPe method. However, it was observed that in the Human Health category, incineration has lower impacts on human toxicity without cancer and higher impacts on human toxicity with cancer.

## 4. Conclusions

The present work allowed us to conclude that CD-5 has lower environmental impacts when analyzed using the ReCiPe, Greenhouse Gas Protocol, and USEtox LCIA methods. On the other hand, in the AWARE method, the most sustainable synthesis was CD-1. Nonetheless, in all the methods, CD-4 has a higher environmental impact.

According to the disposal scenario, for CD-5, it was verified that incineration has slightly lower impacts than the landfill scenario in the ReCiPe and USEtox methods. On the contrary, in the Greenhouse Gas Protocol, the landfill scenario has lower impacts than incineration. In the AWARE method, for CD-1, it was shown that no significant differences exist between both disposal scenarios with landfill being somewhat more sustainable than incineration.

In the ReCiPe method, CD-1 was associated with lower impacts than CD-4 (despite both performing a hydrothermal treatment), which can be explained due to the difference in the synthesis yields. Additionally, in the three analyses (synthesis, incineration, and landfill) performed, it was verified that CD-2 has higher impacts in the Resources category.

In the Greenhouse Gas Protocol, considering all the subcategories, it was noted that the impacts in CD-1 were higher in Biogenic CO_2_ and lower in CO_2_ uptake. Beyond this, Biogenic CO_2_ appears to be a key category in this method, since the environmental impacts were higher in all synthesized CDs.

In the AWARE method, we verified that the synthesis routes where a hydrothermal treatment was performed (CD-1 and CD-4) were either the most sustainable (CD-1) or were responsible for higher impacts (CD-4). This could be explained by the distinct synthesis yields and carbon precursor used in the synthesis or the huge input of water in CD-4. It is worth mentioning that CD-1 was more sustainable in water deprivation.

In USEtox, the analysis of the disposal scenario showed that CD-5 and CD-6 have lower environmental impacts when compared to the remaining syntheses. However, in CD-5 and CD-6, the difference in potential environmental impacts between the two main categories (Human Health and Ecosystems) was higher than in the other syntheses. Beyond this, the impacts of incineration and landfill in CD-1 were higher in Human Health, and ecotoxicity cancer than in the other subcategories. Due to the higher impacts of CD-1 in comparison with the other methods, it was concluded that CD-1 presents less sustainability related to the ecotoxicity of chemicals.

In short, we concluded that, for CD-5, landfill has minor environmental impacts on CO_2_ emissions (Fossil and Biogenic) and incineration has lower impacts in the Human Health and Ecosystems categories (ReCiPe and USEtox methods).

In general, it is possible to conclude that high-yield synthesis (CD-1) is not associated with lower potential impacts than some “standard” bottom-up syntheses, such as CD-5 and CD-6. Actually, in three of the methods used (ReCiPe, Greenhouse Gas Protocol, and USEtox), despite the difference of 12–13% in the synthesis yield between CD-5, CD-6, and CD-1, the impacts were lower for the “standard” bottom-up synthesis. More importantly, in terms of sustainability, CD-1 is not so much “greener” than CD-2 and CD-3, which are “standard” bottom-up syntheses with low yields (CD-2: 7.3% and CD-3: 9.85%). In this sense, it was concluded that current high-yield procedures may not yet be readily-available alternatives for cleaner production, since higher synthesis yields appear to come with higher environmental costs. Nevertheless, the higher impacts associated with higher synthesis yields appear to come from increased consumption of electricity associated with the hydrothermal and drying processes. Thus, if electricity consumption could be reduced in those steps, these new strategies could indeed be more suitable choices for producing CDs in a more environmentally friendly way.

## Figures and Tables

**Table 1:** Synthesis yield of each CD under study.

CDs	Synthesis Yield (wt. %)	References
CD-1	40.1	[36]
CD-2	7.3	[33]
CD-3	9.85	[27]
CD-4	1.8	[29]
CD-5	28.5	[29]
CD-6	26.9	[29]

## Supplementary Materials

The following supporting information can be downloaded at: https://www.mdpi.com/article/10.3390/ma15103446/s1, Table S1. Inventory of raw materials for each CD under study, in which quantities are referred to as 1 kg of CD; Figure S1. Relative environmental impacts of the synthesis under study applying the ReCiPe endpoint method. (A) CD-1; (B) CD-2, (C) CD-3; (D) CD-4, (E) CD-5 and (F) CD-6. The abbreviations are explained in Section 2.4; Figure S2. Relative environmental impacts of the synthesis under study and the disposal scenario of incineration, applying the ReCiPe endpoint method. (A) CD-1; (B) CD-2, (C) CD-3; (D) CD-4, (E) CD-5 and (F) CD-6. The abbreviations are explained in Section 2.4; Figure S3. Relative environmental impacts of the synthesis under study and the disposal scenario of landfill, applying the ReCiPe endpoint method. (A) CD-1, (B) CD-2, (C) CD-3, (D) CD-4, (E) CD-5, and (F) CD-6. The abbreviations are explained in Section 2.4; Figure S4. Relative environmental impacts of CD-5 for incineration and landfill disposal scenario, applying the ReCiPe method; Figure S5. Relative environmental impacts of syntheses under study, applying the Greenhouse Gas Protocol method. (A) CD-1, (B) CD-2, (C) CD-3, (D) CD-4, (E) CD-5, and (F) CD-6; Figure S6. Relative environmental impacts of syntheses under study for the incineration disposal scenario, applying the Greenhouse Gas Protocol method. (A) CD-1, (B) CD-2, (C) CD-3, (D) CD-4, (E) CD-5, and (F) CD-6; Figure S7. Relative environmental impacts of syntheses under study for the landfill disposal scenario, applying the Greenhouse Gas Protocol method. (A) CD-1, (B) CD-2, (C) CD-3, (D) CD-4, (E) CD-5, and (F) CD-6; Figure S8. Relative environmental impacts of syntheses under study, applying the AWARE method. (A) CD-1, (B) CD-2, (C) CD-3, (D) CD-4, (E) CD-5, and (F) CD-6; Figure S9. Relative environmental impacts of syntheses under study for the incineration disposal scenario, applying the AWARE method. (A) CD-1, (B) CD-2, (C) CD-3; (D) CD-4, (E) CD-5 and (F) CD-6; Figure S10. Relative environmental impacts of syntheses under study for the landfill disposal scenario, applying the AWARE method. (A) CD-1, (B) CD-2, (C) CD-3, (D) CD-4, (E) CD-5, and (F) CD-6; Figure S11. Relative environmental impacts of syntheses under study, applying the USEtox method. (A) CD-1, (B) CD-2, (C) CD-3, (D) CD-4, (E) CD-5, and (F) CD-6; Figure S12. Relative environmental impacts of syntheses under study, for the incineration disposal scenario, applying the USEtox method. (A) CD-1, (B) CD-2, (C) CD-3, (D) CD-4, (E) CD-5, and (F) CD-6; Figure S13. Relative environmental impacts of syntheses under study, for the landfill disposal scenario, applying the USEtox method. (A) CD-1, (B) CD-2, (C) CD-3, (D) CD-4, (E) CD-5, and (F) CD-6.
