# Supplementary material for: Life Cycle Assessment-Based Comparative Study between High-Yield and “Standard” Bottom-Up Procedures for the Fabrication of Carbon Dots
Source: Materials (Basel). 2022 May 11;15(10):3446. doi: 10.3390/ma15103446 (PMC9145381; doi:10.3390/ma15103446)
Supplement: Supplementary file 1 [file materials-15-03446-s001.zip › materials-1680133-supplementary.pdf]

Supplementary Materials

Table S1. Inventory of raw materials for each CD under study, in which quantities are referred to 1 kg of CD.

Ecoinvent® 3.5 database	CD-1	CD-2	CD-3	CD-4	CD-5	CD-6
Glucose {GLO}∣market for glucose∣ APOS, S	2.49 kg					
Sodium hydroxide, without water, in 50% solution state {GLO}∣market for∣ APOS, S	1.49 kg					
Hydrogen peroxide, without water, in 50% solution state {GLO}∣market for∣ APOS, S	0.239 kg					
Citric Acid {GLO}∣market for∣ APOS, S		7.2 kg	7.61 kg	50 kg	2.4 kg	3 kg
Ethylenediamine {RER}∣ market for∣ APOS, S		7.2 kg				
Urea, as N {GLO}∣ market for∣ APOS, S			2.54 kg	16 kg	0.8 kg	1 kg
Water, deionized, from tap water, at user {Europe without Switzerland}∣ market for water, deionized, from tap water, at user∣ APOS, S	24.9 kg	77.9 kg	101.5 kg	330 kg	20 kg	20 kg
Electricity, medium voltage {RER}∣market group for∣ APOS, S	146.8 kWh	24.2 kWh	46.09 kWh	17 kWh	12 kWh	17 kWh

ReCiPe method

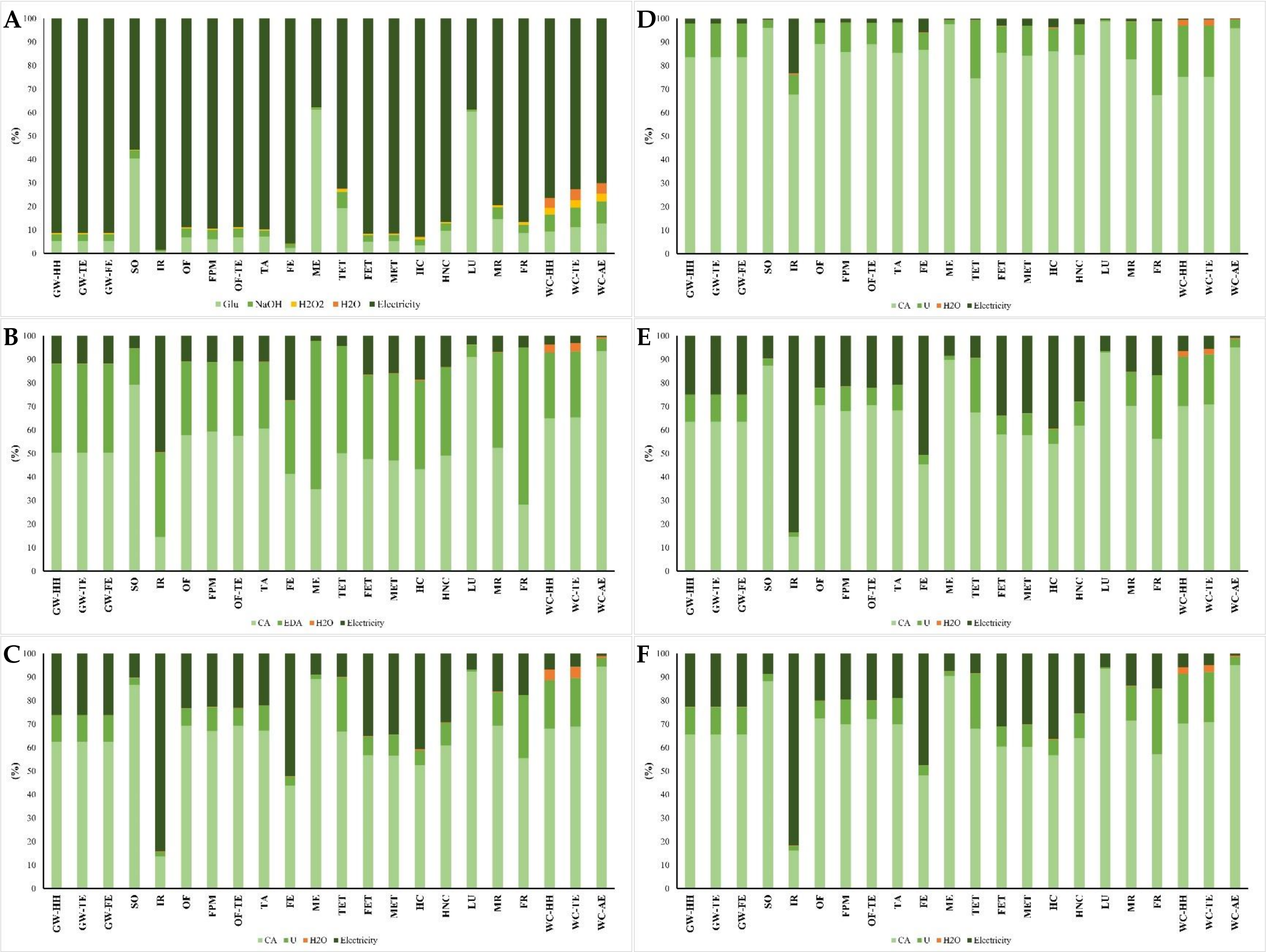

Figure S1. Relative environmental impacts of the synthesis under study applying ReCiPe endpoint method. (**A**) CD-1; (**B**) CD-2, (**C**) CD-3; (**D**) CD-4, (**E**) CD-5 and (**F**) CD-6. The abbreviations are explained in Section 2.4.

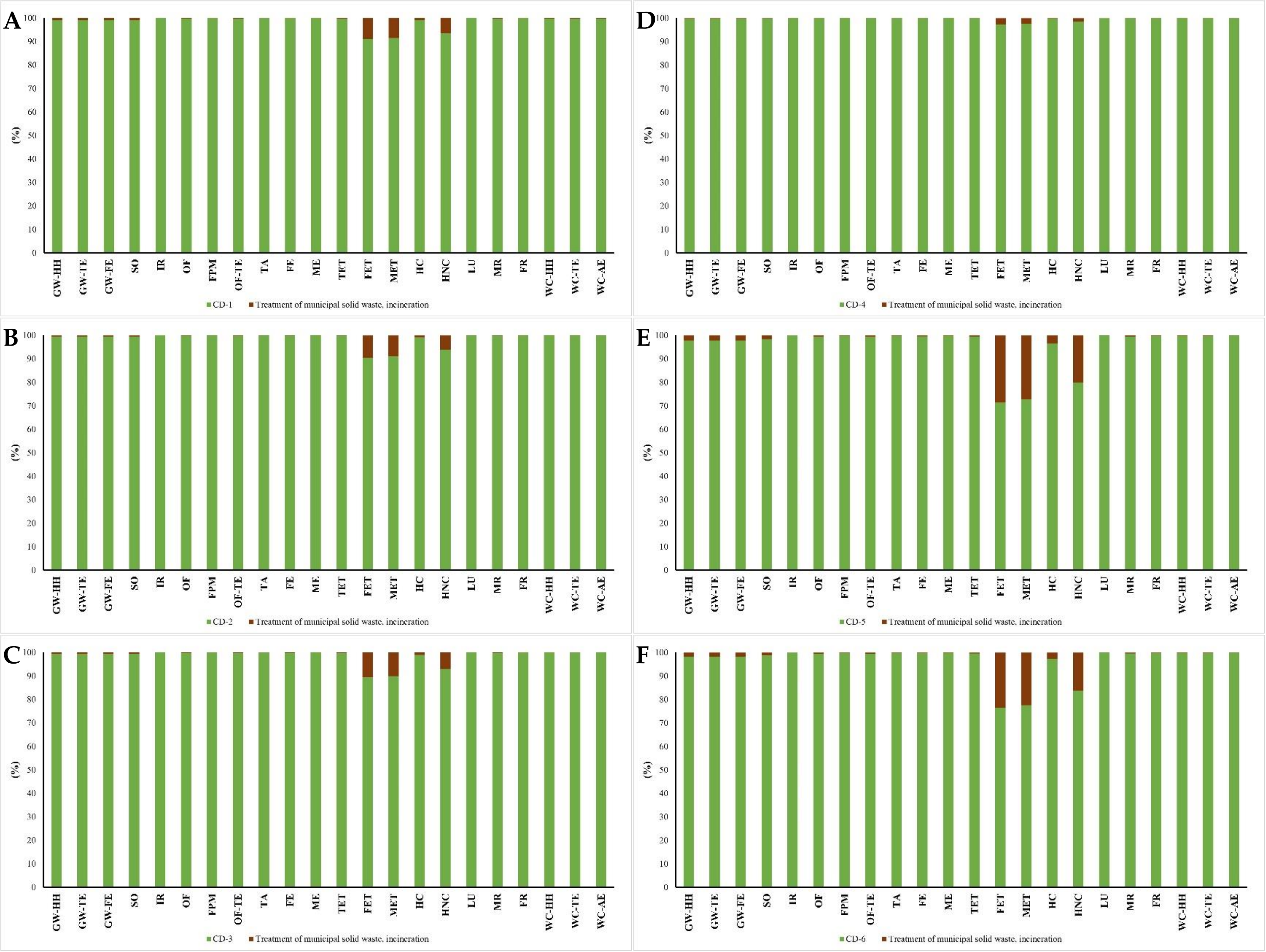

Figure S2. Relative environmental impacts of the synthesis under study and the disposal scenario of incineration, applying ReCiPe endpoint method. (**A**) CD-1; (**B**) CD-2, (**C**) CD-3; (**D**) CD-4, (**E**) CD-5 and (**F**) CD-6. The abbreviations are explained in Section 2.4.

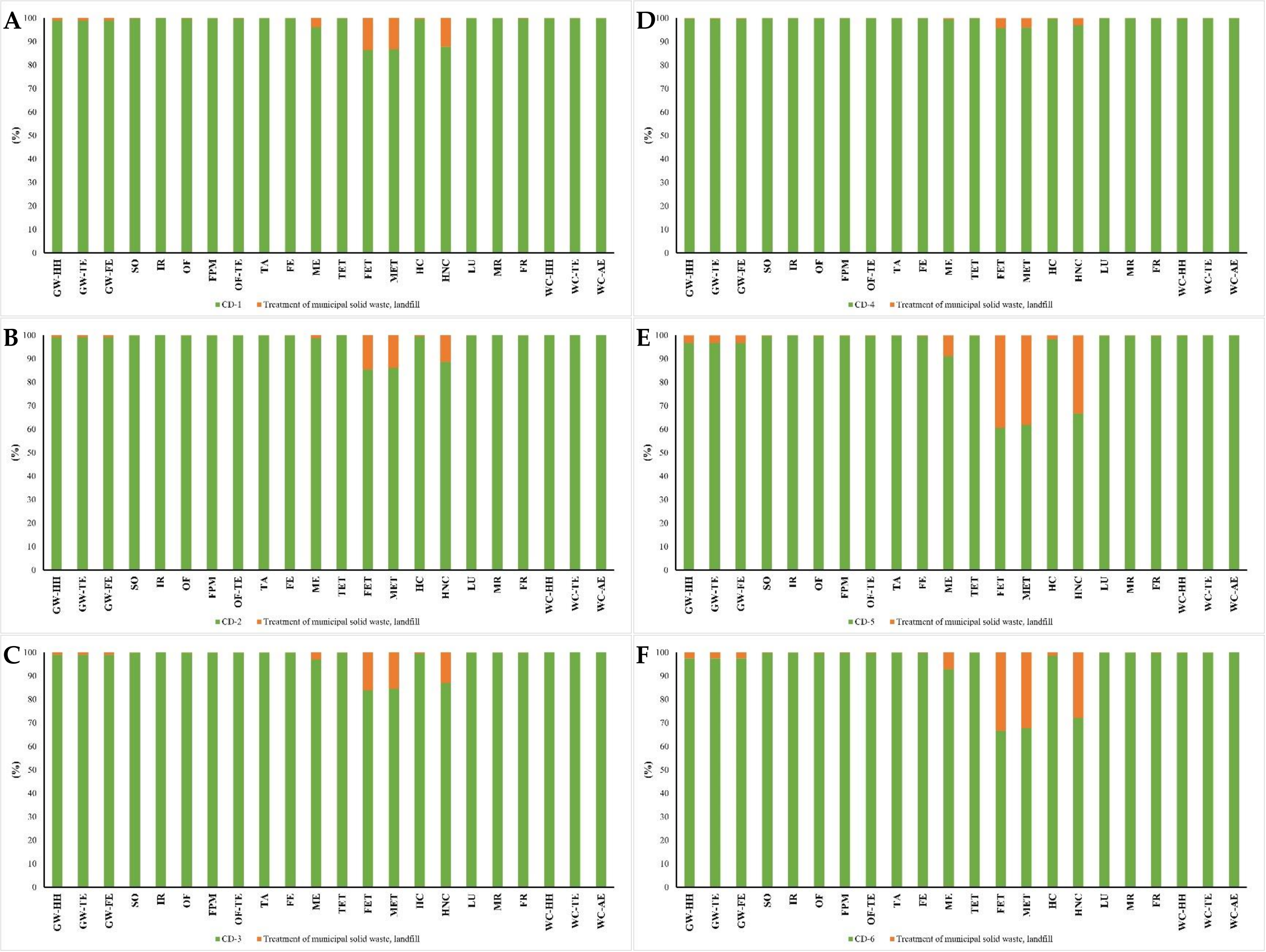

Figure S3. Relative environmental impacts of the synthesis under study and the disposal scenario of landfill, applying ReCiPe endpoint method. (**A**) CD-1; (**B**) CD-2, (**C**) CD-3; (**D**) CD-4, (**E**) CD-5 and (**F**) CD-6. The abbreviations are explained in Section 2.4

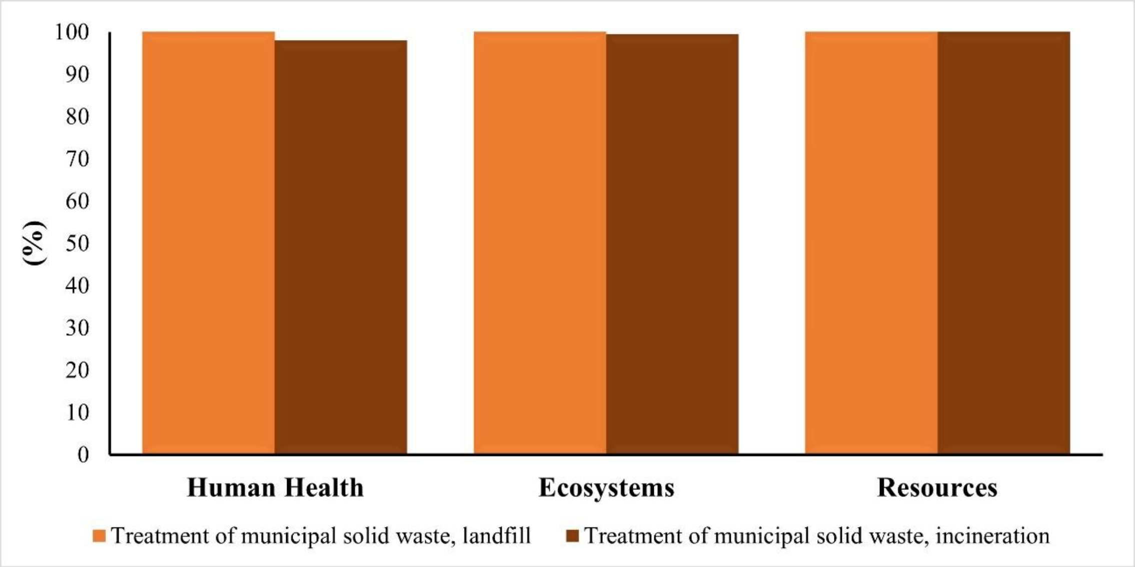

Figure S4. Relative environmental impacts of CD-5 for incineration and landfill disposal scenario, applying ReCiPe method.

Greenhouse Gas Protocol method

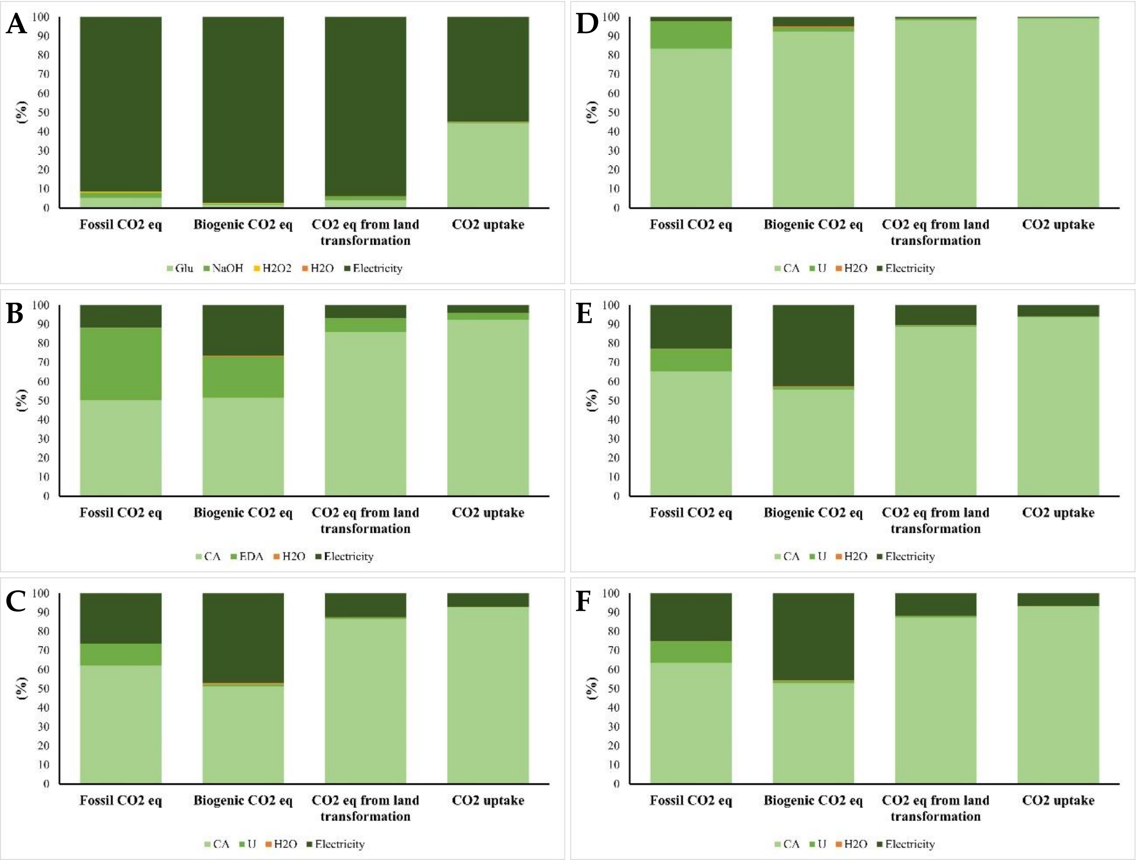

Figure S5. Relative environmental impacts of syntheses under study, applying Greenhouse Gas Protocol method. (**A**) CD-1; (**B**) CD-2, (**C**) CD-3; (**D**) CD-4, (**E**) CD-5 and (**F**) CD-6.

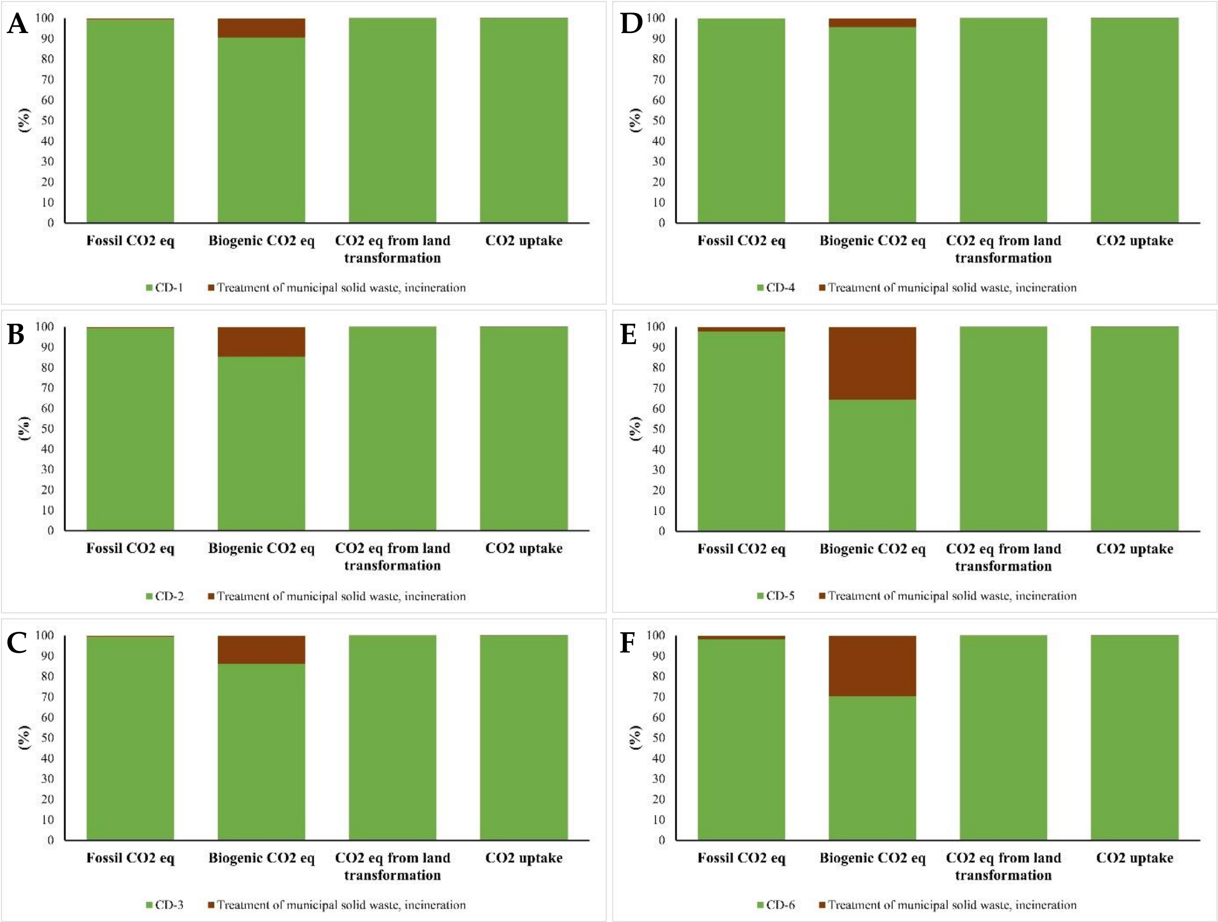

Figure S6. Relative environmental impacts of syntheses under study for incineration disposal scenario, applying Greenhouse Gas Protocol method. (**A**) CD-1; (**B**) CD-2, (**C**) CD-3; (**D**) CD-4, (**E**) CD-5 and (**F**) CD-6.

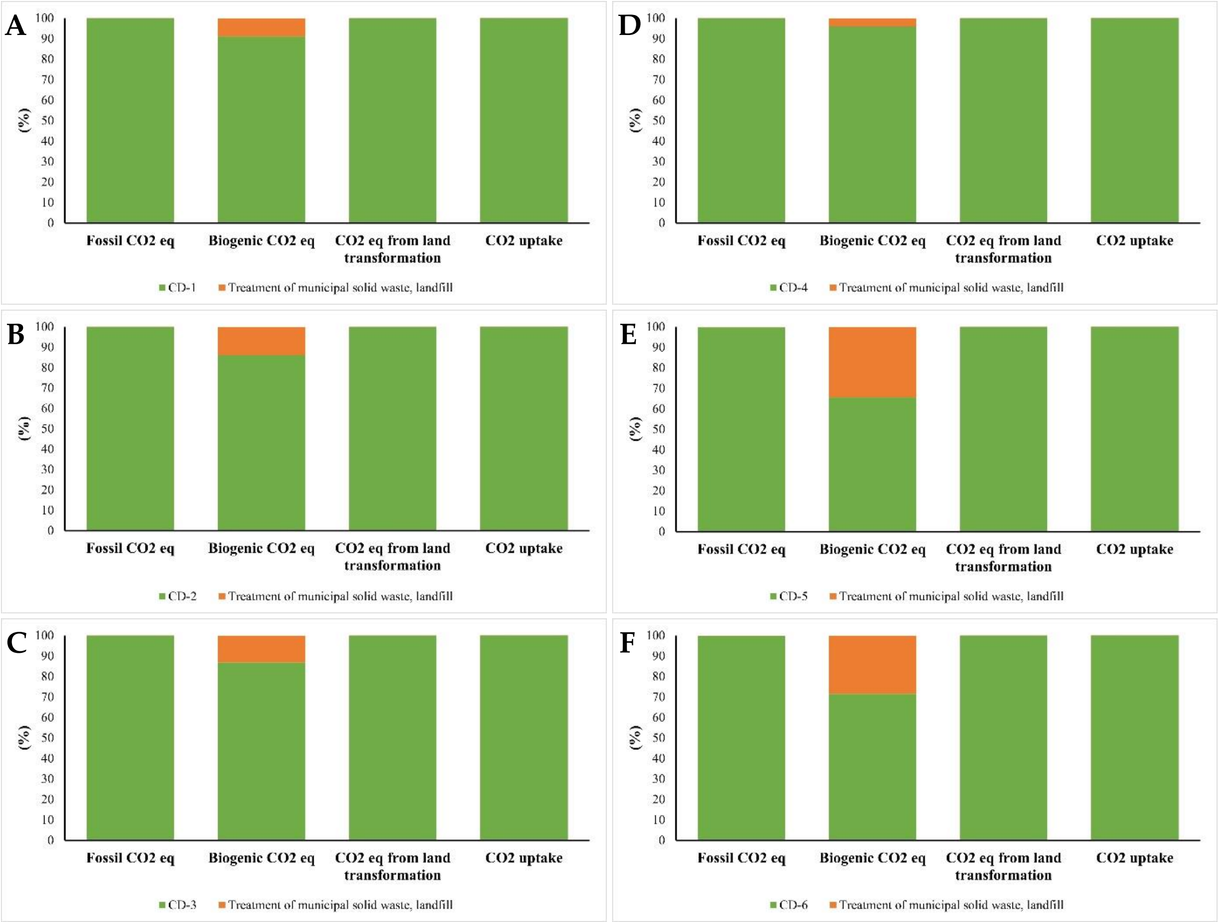

Figure S7. Relative environmental impacts of syntheses under study for landfill disposal scenario, applying Greenhouse Gas Protocol method. (**A**) CD-1; (**B**) CD-2, (**C**) CD-3; (**D**) CD-4, (**E**) CD-5 and (**F**) CD-6.

AWARE method

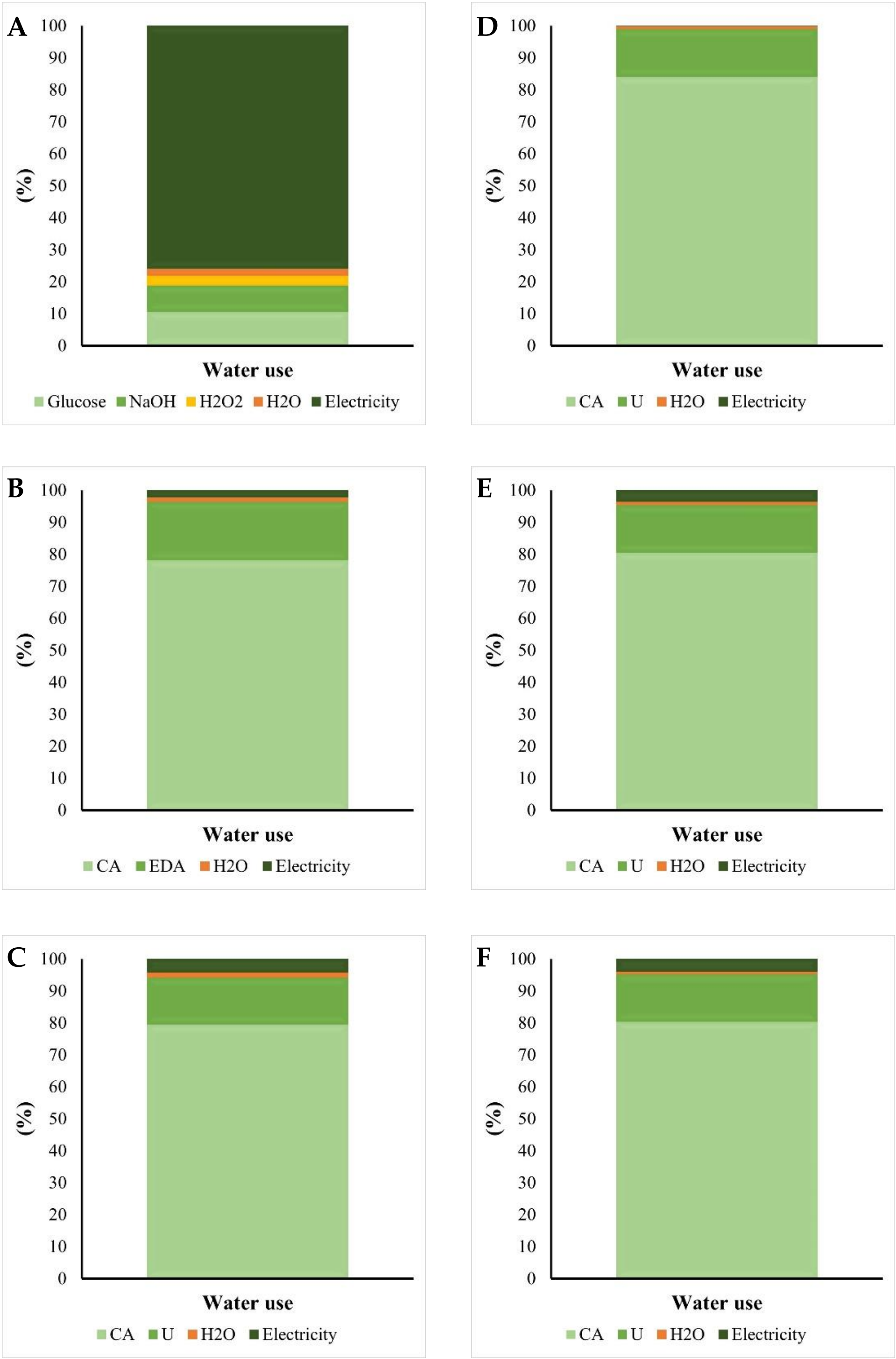

Figure S8. Relative environmental impacts of syntheses under, applying AWARE method. (**A**) CD-1; (**B**) CD-2, (**C**) CD-3; (**D**) CD-4, (**E**) CD-5 and (**F**) CD-6.

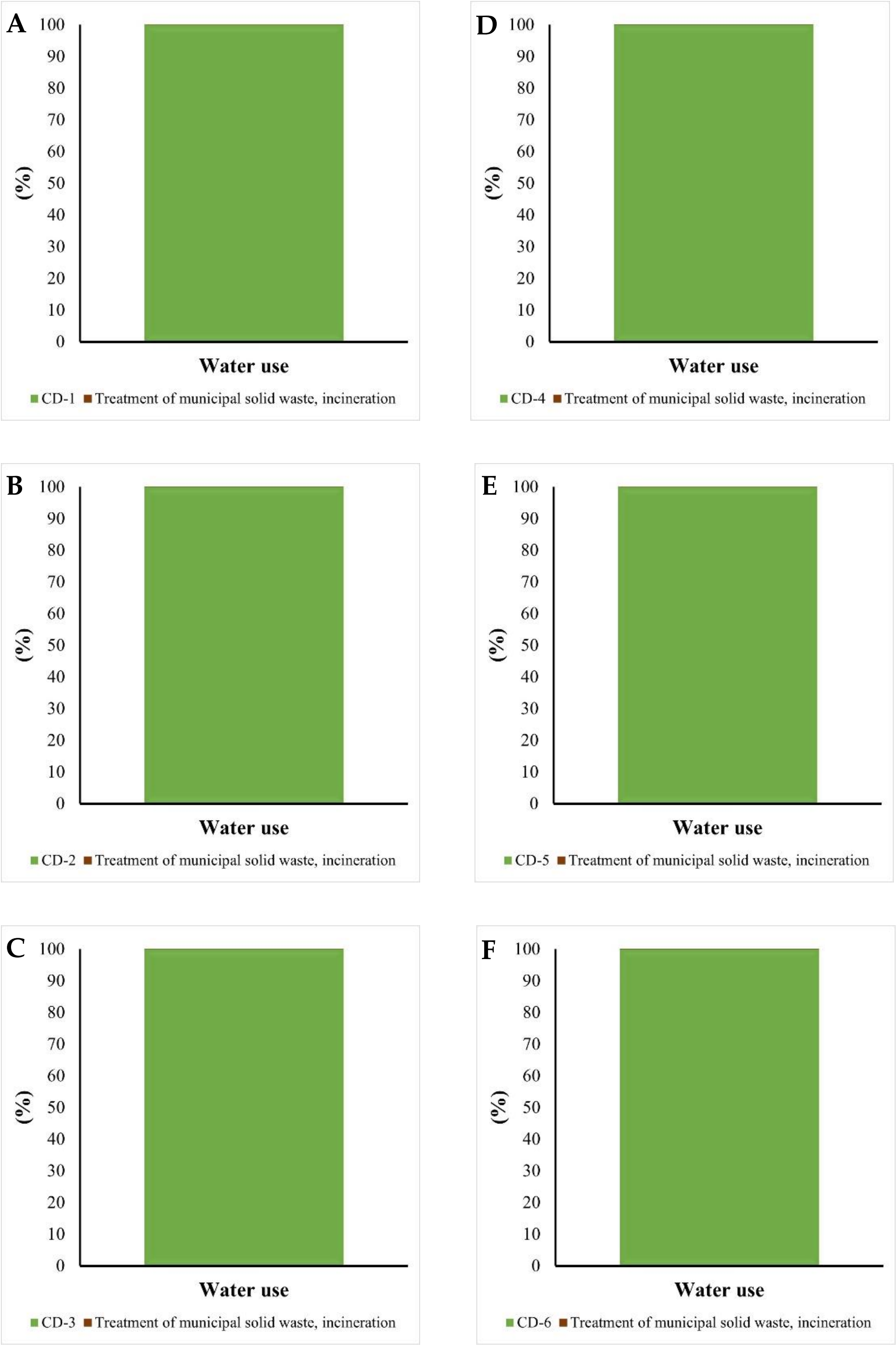

Figure S9. Relative environmental impacts of syntheses under for incineration disposal scenario, applying AWARE method. (**A**) CD-1; (**B**) CD-2, (**C**) CD-3; (**D**) CD-4, (**E**) CD-5 and (**F**) CD-6.

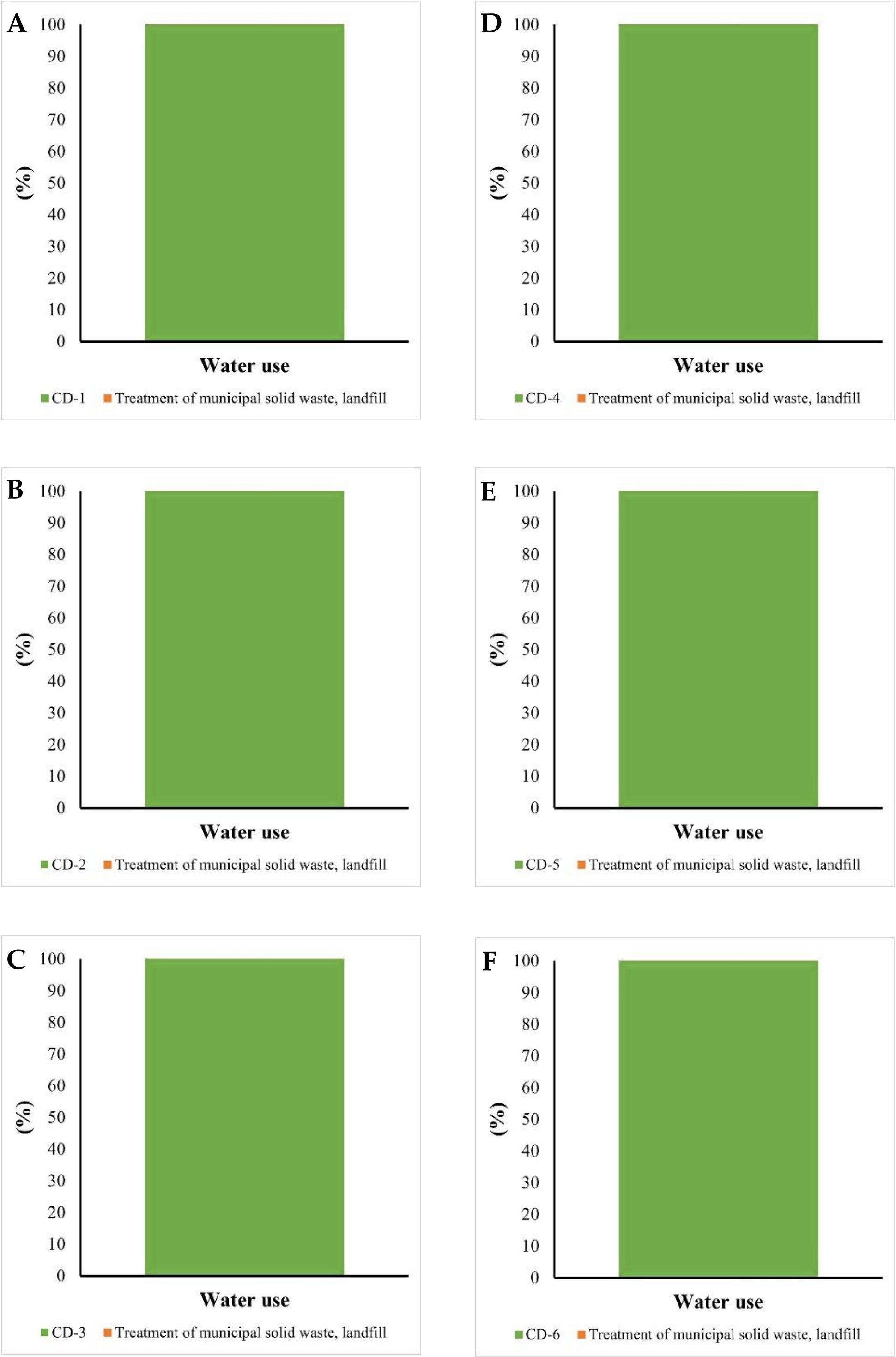

Figure S10. Relative environmental impacts of syntheses under for landfill disposal scenario, applying AWARE method. (**A**) CD-1; (**B**) CD-2, (**C**) CD-3; (**D**) CD-4, (**E**) CD-5 and (**F**) CD-6.

USEtox method

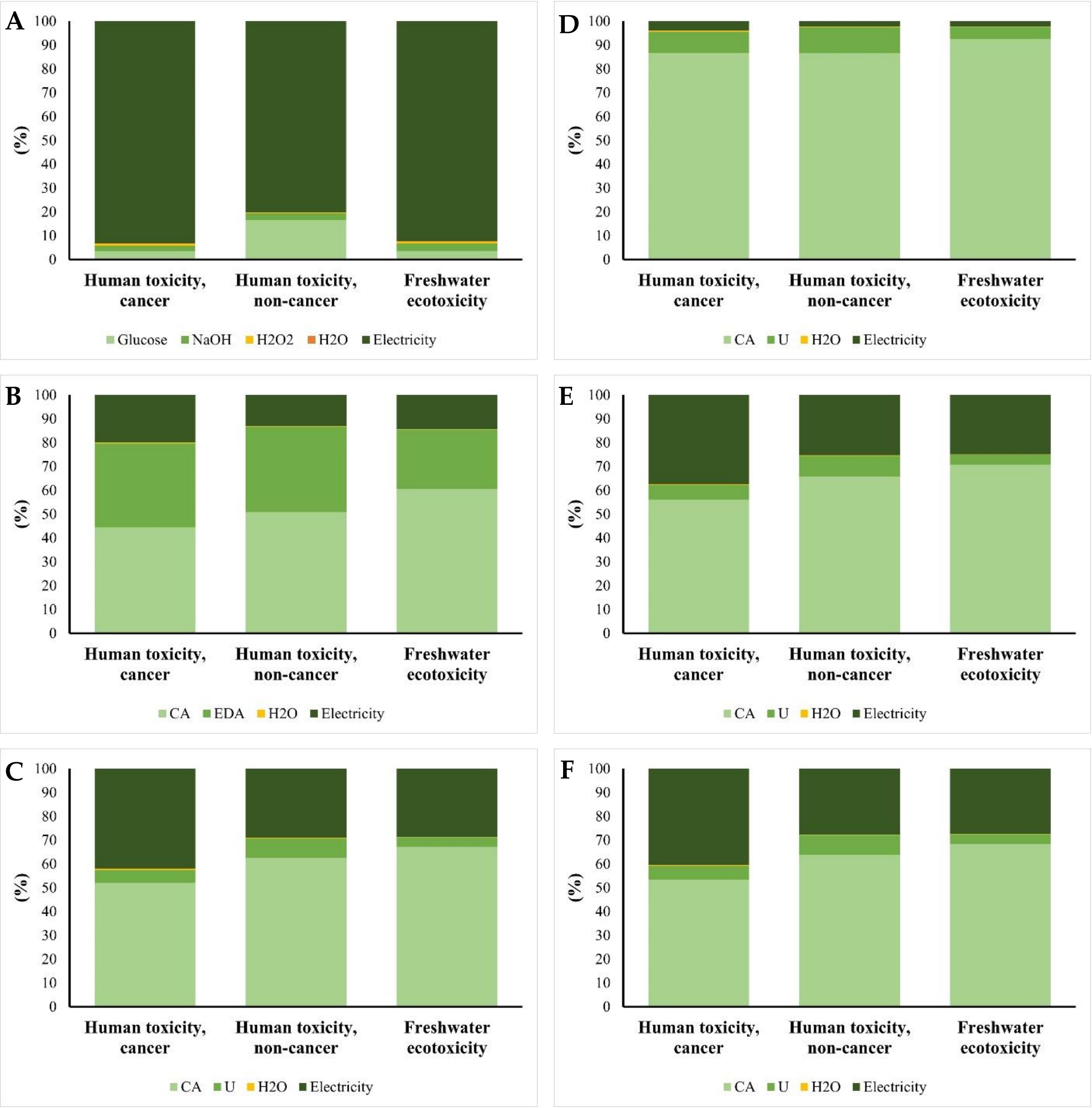

Figure S11. Relative environmental impacts of syntheses under, applying USEtox method. (**A**) CD-1; (**B**) CD-2, (**C**) CD-3; (**D**) CD-4, (**E**) CD-5 and (**F**) CD-6.

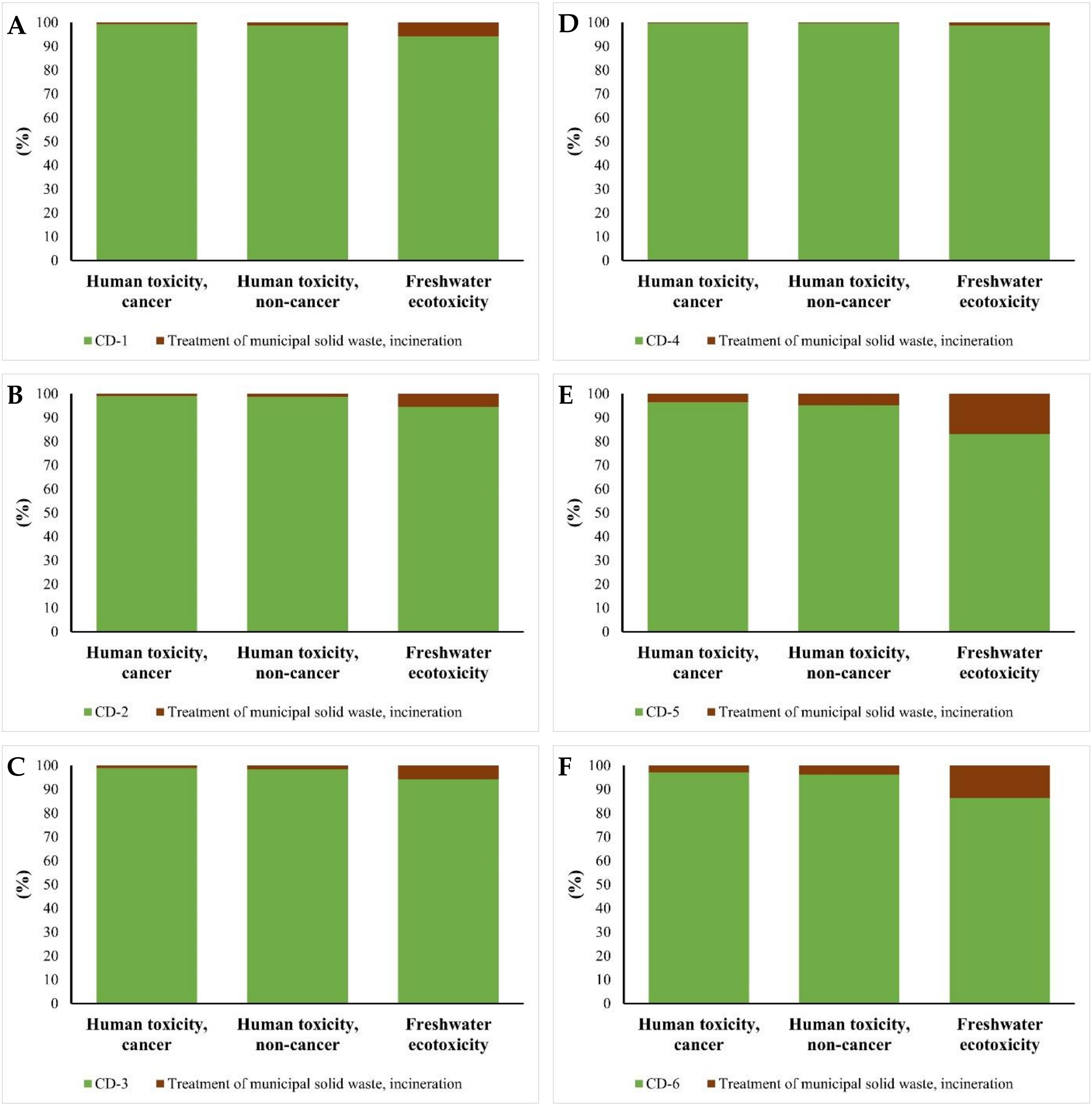

Figure S12. Relative environmental impacts of syntheses under for incineration disposal scenario, applying USEtox method. (**A**) CD-1; (**B**) CD-2, (**C**) CD-3; (**D**) CD-4, (**E**) CD-5 and (**F**) CD-6.

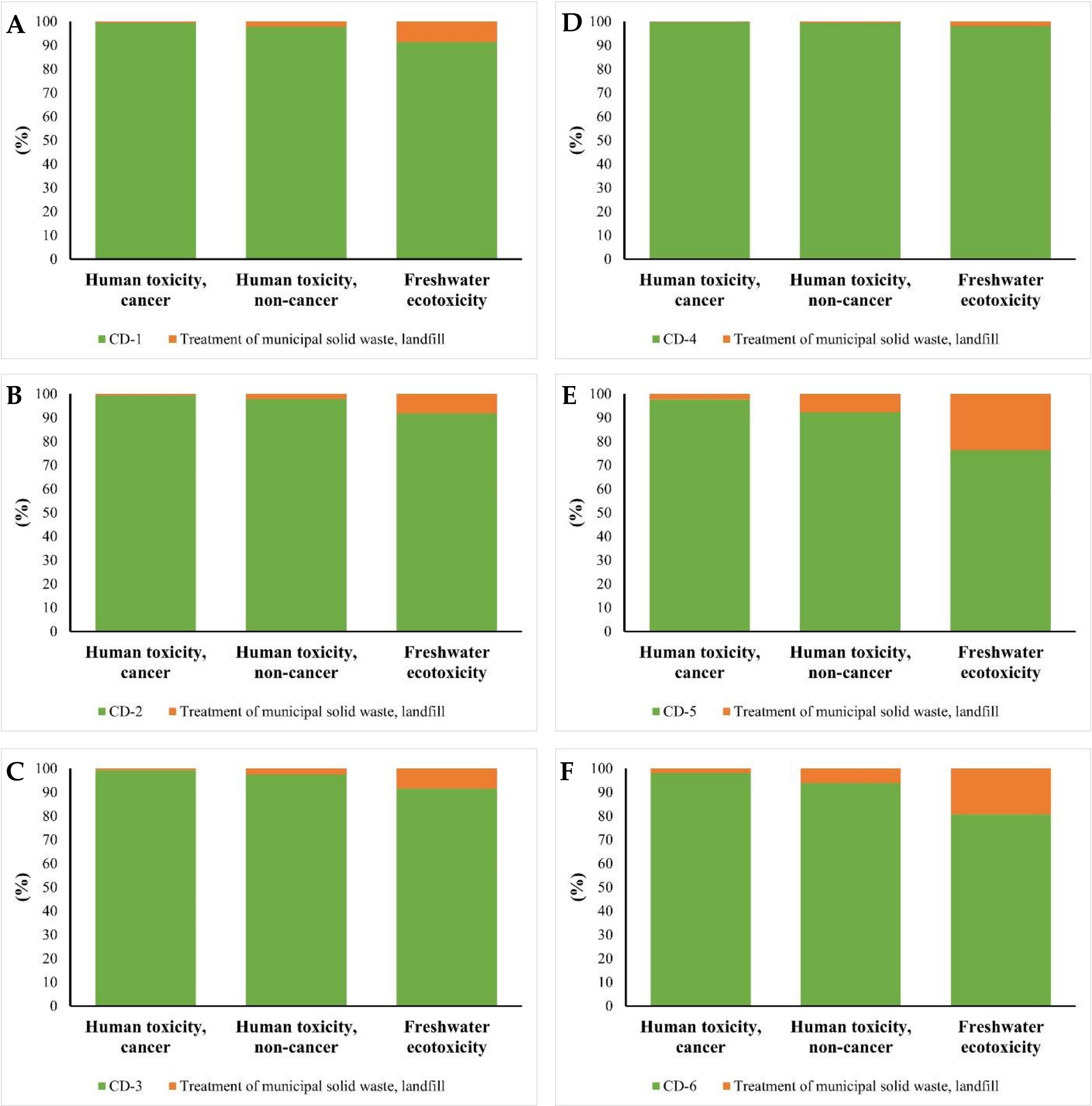

Figure S13. Relative environmental impacts of syntheses under for landfill disposal scenario, applying USEtox method. (**A**) CD-1; (**B**) CD-2, (**C**) CD-3; (**D**) CD-4, (**E**) CD-5 and (**F**) CD-6.
